# Endothelial FOXM1 and Dab2 promote diabetic wound healing

**DOI:** 10.1172/jci.insight.186504

**Published:** 2025-01-23

**Authors:** Sudarshan Bhattacharjee, Jianing Gao, Yao Wei Lu, Shahram Eisa-Beygi, Hao Wu, Kathryn Li, Amy E. Birsner, Scott Wong, Yudong Song, John Y-J. Shyy, Douglas B. Cowan, Wendong Huang, Wenyi Wei, Masanori Aikawa, Jinjun Shi, Hong Chen

**Affiliations:** 1 Vascular Biology Program, Boston Children’s Hospital, Boston, Massachusetts, USA.; 2 Department of Surgery and; 3 Department of Ophthalmology, Harvard Medical School, Boston, Massachusetts, USA.; 4 Center for Nanomedicine and Department of Anesthesiology, Perioperative and Pain Medicine, Brigham and Women’s Hospital, Harvard Medical School, Boston, Massachusetts, USA.; 5 Division of Cardiovascular Medicine, Department of Medicine, University of California, San Diego, La Jolla, California, USA.; 6 Division of Molecular Diabetes Research, Department of Diabetes and Metabolic Diseases, City of Hope National Medical Center, Duarte, California, USA.; 7 Department of Pathology, Beth Israel Deaconess Medical Center, and; 8 Center for Interdisciplinary Cardiovascular Sciences, Cardiovascular Division, Department of Medicine, Brigham and Women’s Hospital, Harvard Medical School, Boston, Massachusetts, USA.

**Keywords:** Angiogenesis, Adaptor proteins

## Abstract

Diabetes mellitus can cause impaired and delayed wound healing, leading to lower extremity amputations; however, the mechanisms underlying the regulation of vascular endothelial growth factor–dependent (VEGF-dependent) angiogenesis remain unclear. In our study, the molecular underpinnings of endothelial dysfunction in diabetes are investigated, focusing on the roles of disabled-2 (Dab2) and Forkhead box M1 (FOXM1) in VEGF receptor 2 (VEGFR2) signaling and endothelial cell function. Bulk RNA-sequencing analysis identified significant downregulation of Dab2 in high-glucose-treated primary mouse skin endothelial cells. In diabetic mice with endothelial deficiency of Dab2, in vivo and in vitro angiogenesis and wound healing were reduced when compared with wild-type diabetic mice. Restoration of Dab2 expression by injected mRNA-containing, LyP-1–conjugated lipid nanoparticles rescued impaired angiogenesis and wound healing in diabetic mice. Furthermore, FOXM1 was downregulated in skin endothelial cells under high-glucose conditions as determined by RNA-sequencing analysis. FOXM1 was found to bind to the Dab2 promoter, regulating its expression and influencing VEGFR2 signaling. The FOXM1 inhibitor FDI-6 reduced Dab2 expression and phosphorylation of VEGFR2. Our study provides evidence of the crucial roles of Dab2 and FOXM1 in diabetic endothelial dysfunction and establishes targeted delivery as a promising treatment for diabetic vascular complications.

## Abstract 2

We show the endocytic adaptor Dab2 plays a critical role in blood vessel formation in diabetes, which may lead to new treatments for wound healing.

## Graphical abstract

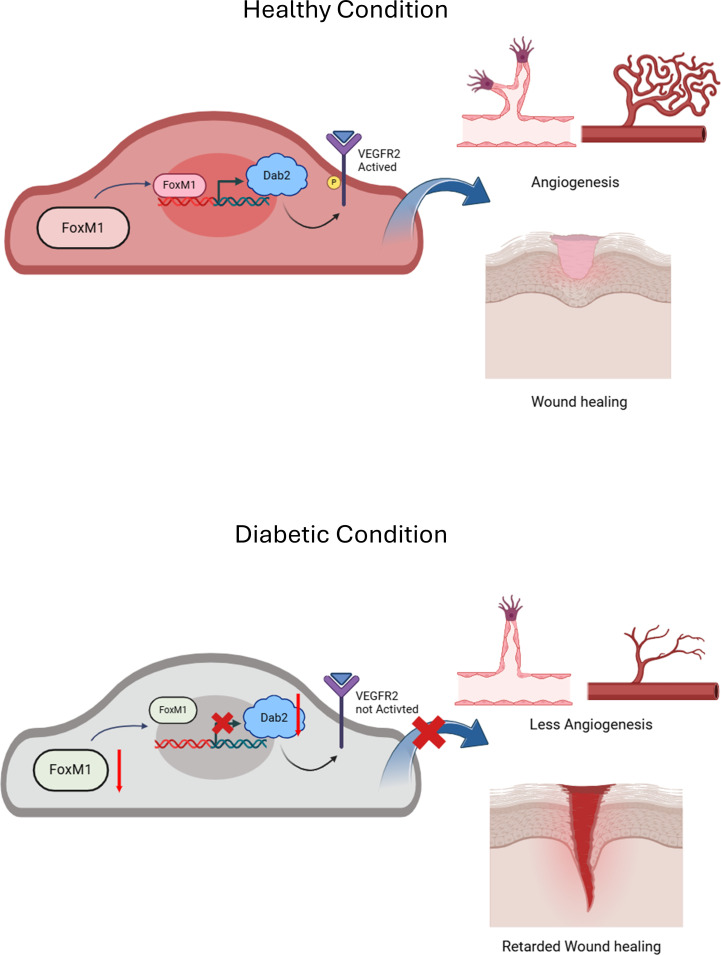

## Introduction

One of the most serious pathological outcomes of diabetes mellitus is impaired or delayed wound healing, which — in severe cases — can lead to lower extremity amputations (1–3). Although the etiological basis of chronic nonhealing wounds is multifaceted, aberrant angiogenesis is, at least in part, involved in sustaining this phenotype. During wound healing, angiogenic sprouts descend upon the wound area to establish normoxia, and they eventually fashion a microvascular network to restore oxygen and nutrient delivery to the wound area and help remove debris (4–6). Therefore, promoting angiogenesis is crucial for wound healing, and developing effective targets for angiogenesis could benefit millions of patients with diabetes.

Vascular endothelial growth factor (VEGF) is a critical angiogenic factor that signals through VEGF receptors (VEGFRs) (7). Among the family of VEGFRs, VEGFR2 potentiates angiogenesis more potently than other VEGFRs. Binding of VEGF to VEGFR2 leads to the phosphorylation of VEGFR2 and activation of downstream signaling pathways, including mitogen-activated protein kinase/extracellular signal–regulated kinase (MAPK/ERK) and phosphatidylinositol-3-kinase/v-akt murine thymoma viral oncogene homolog 1 (PI3K/AKT), which promote endothelial cell (EC) proliferation, migration, and survival (8, 9). In diabetic conditions, there is a decrease in VEGF-induced phosphorylation of VEGFR2 and downstream signaling, leading to impaired angiogenesis (10–12). Hence, gaining insights into the regulation of VEGFR2-dependent angiogenesis may lead to the identification of new therapeutic strategies in this context.

Several studies have shown that the highly conserved adaptor protein disabled-2 (Dab2) plays a direct role in regulating VEGF signaling in ECs (13, 14). Dab2 is involved in the regulation of endocytosis and lysosomal degradation of receptor tyrosine kinases (RTKs), including VEGFR2. Dab2 binds to the cytoplasmic tail of VEGFR2 and promotes its endocytosis and recycling (14), thereby serving to enhance VEGFR2-mediated angiogenesis. In spite of this, the specific structural domain through which Dab2 interacts with VEGFR2 is uncertain. At the same time, the molecular mechanisms underlying Dab2-mediated angiogenesis, particularly in the context of wound healing in diabetes, are not clear. Identifying factors that regulate *Dab2* transcription is key to uncovering the mechanisms of Dab2’s role in EC angiogenesis under diabetic conditions. More pressingly, it remains unclear if modifying Dab2 levels could serve as a therapeutic strategy to enhance diabetic wound healing. To better target Dab2, it is essential to develop exogenous supplementation methods that have a shorter half-life and higher efficiency.

The present study was designed to dissect the potential involvement of Dab2 in regulating VEGF signaling during angiogenesis in the context of wound healing in diabetes. Using an EC-specific Dab2-knockout mouse model, we found that Forkhead box M1 (FOXM1) regulates Dab2 expression in ECs and promotes diabetic wound healing. This transcription factor orchestrates the expression of genes essential for cell cycle progression, thus facilitating cell growth and division, a process that is vital for tissue repair and regeneration (15–18). We found that FOXM1 positively regulates Dab2 expression by directly binding to its promoter to influence transcription and protein levels of Dab2. By injecting *Dab2* mRNA encapsulated in lipid nanoparticles (LNPs), or using the FOXM1 inhibitor FDI-6, wound healing was significantly enhanced through increased angiogenesis, which could lay the foundation for the development of novel therapies to enhance angiogenesis in diabetes.

Together, our results suggest that Dab2 plays a critical role in VEGF signaling and angiogenesis in ECs under diabetic conditions by regulating the activation of VEGFR2. We also identified the specific binding domain of *Dab2* that binds to VEGFR2 and demonstrated that FOXM1 regulated transcription of this adaptor protein in ECs. Our findings indicated that Dab2 may represent a previously unidentified potential target for improving diabetic wound healing.

## Results

### Diabetes and high-glucose treatment in ECs lead to the downregulation of Dab2.

**Figure 1:**
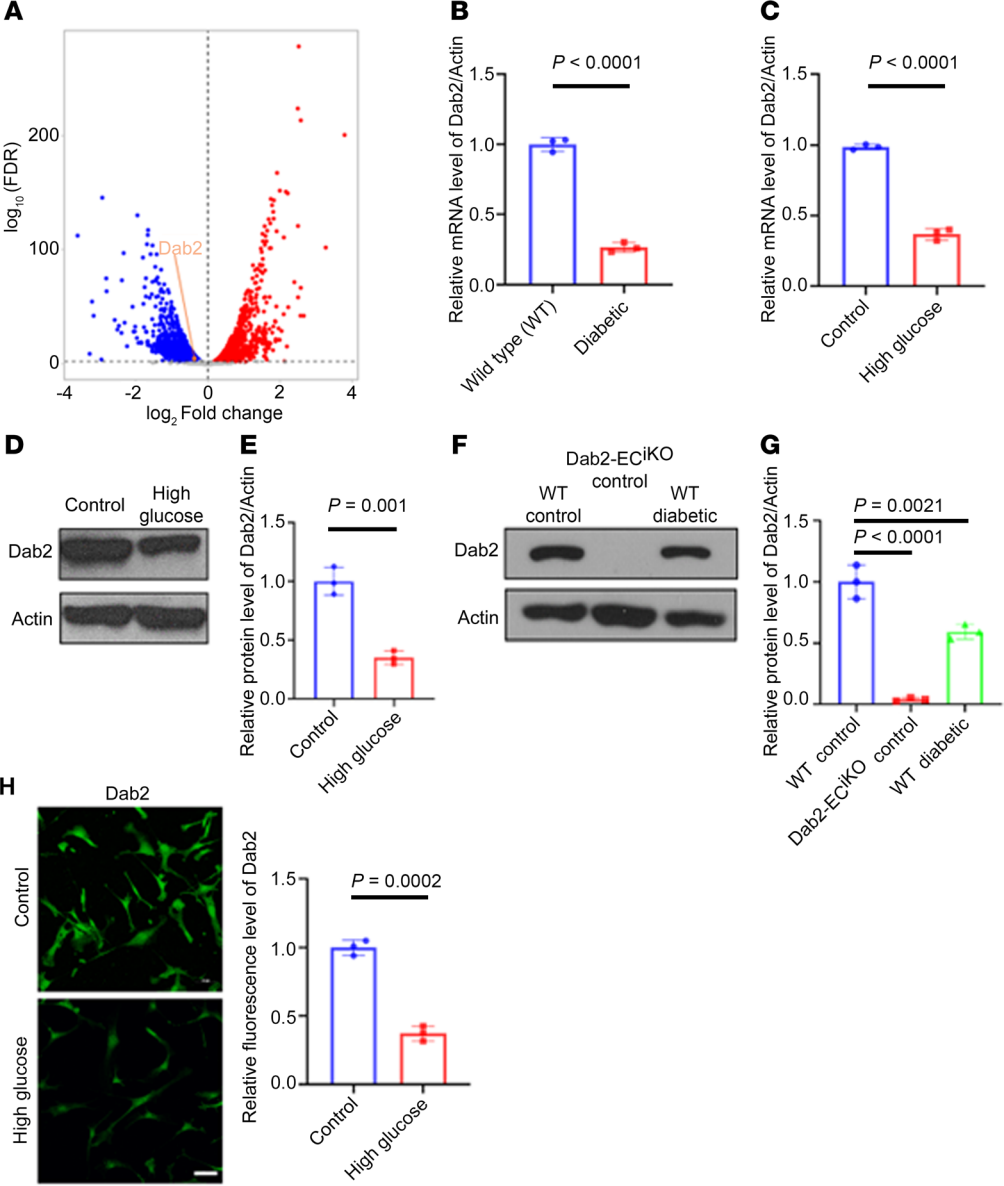
Diabetes and high-glucose treatment in ECs lead to the downregulation of Dab2. (**A**) Volcano plot of differentially expressed genes and in skin ECs cultured in high versus normal concentration of glucose for 48 hours. The *x* axis shows the log_2_ fold-change (log_2_FC), and the *y* axis represents the negative logarithm of the false discovery rate (–log_10_ FDR) (*n* = 3 per group of mice). (**B**) RNA abundance of *Dab2* in ECs isolated from WT or diabetic mouse skin determined by quantitative reverse transcriptase PCR (qRT-PCR or qPCR) (*n* = 3 cell repetitions, results are presented as mean ± SD, *P* value calculated by *t* test). (**C**) RNA abundance of *Dab2* in skin ECs cultured in normal or high concentration of glucose determined by qRT-PCR (*n* = 3 cell repetitions, results are presented as mean ± SD, *P* value calculated by *t* test). (**D**) Representative Western blots of Dab2 in skin ECs cultured in normal (Control) or high concentration of glucose. (**E**) Quantitation of protein level of Dab2 relative to Actin in **C** (*n* = 3 cell repetitions, results are presented as mean ± SD, *P* value calculated by *t* test). (**F**) Representative Western blots of Dab2 in skin ECs isolated from WT control mice, Dab2-EC^iKO^ control mice, and WT diabetic mice. (**G**) Quantitation of protein level of Dab2 relative to Actin in **E** (*n* = 3 cell repetitions, results are presented as mean ± SD, *P* value calculated by ANOVA). (**H**) Representative immunofluorescence staining of Dab2 (shown in green) in ECs treated with high or normal concentration of glucose for 48 hours. Scale bar = 50 μm. (**I**) Quantitation of fluorescence intensity in **H** (*n* = 3 cell repetitions, results are presented as mean ± SD, *P* value calculated by *t* test).

Given the crucial roles in both physiological and pathological angiogenesis, we sought to determine if endocytic adaptor proteins were also involved in mitigating aspects of diabetic angiogenesis. To address this goal, we isolated cluster of differentiation 31–enriched (CD31-enriched) primary mouse ECs and treated them with normal (5 mmol/L) or high glucose concentrations (20 mmol/L), a condition mimicking hyperglycemia in diabetes mellitus, for a period of 48 hours, followed by bulk RNA-sequencing analysis. Differential gene expression analysis revealed 168 significantly downregulated and 386 significantly upregulated genes in the ECs grown in high-glucose culture conditions compared with ECs cultured in normal-glucose medium (Figure 1A and Supplemental Figure 1; supplemental material available online with this article; https://doi.org/10.1172/jci.insight.186504DS1). Volcano plot analysis revealed downregulation of *Dab2* mRNA levels in high-glucose-treated ECs compared with the control group (Figure 1A). Further corroborating these findings, quantitative PCR (qPCR) analysis showed that high-glucose treatment or ECs from diabetic mice resulted in the downregulation of Dab2 (Figure 1, B and C).

Consistent with the RNA results, Western blot analysis revealed significantly downregulated Dab2 protein levels in the diabetic group or high-glucose-treated group compared with nondiabetic controls (Figure 1, D–G). Downregulation of Dab2 expression in diabetic conditions was also verified by immunostaining of skin ECs cultured in normal or high-glucose concentrations (Figure 1, H and I). Together, these in vitro observations suggest that hyperglycemic ECs exhibit downregulation of *Dab2* mRNA and protein levels, along with reduced expression of genes involved in cellular metabolism, growth, and proliferation.

### EC-specific Dab2 knockout causes reduced angiogenesis in vivo.

**Figure 2:**
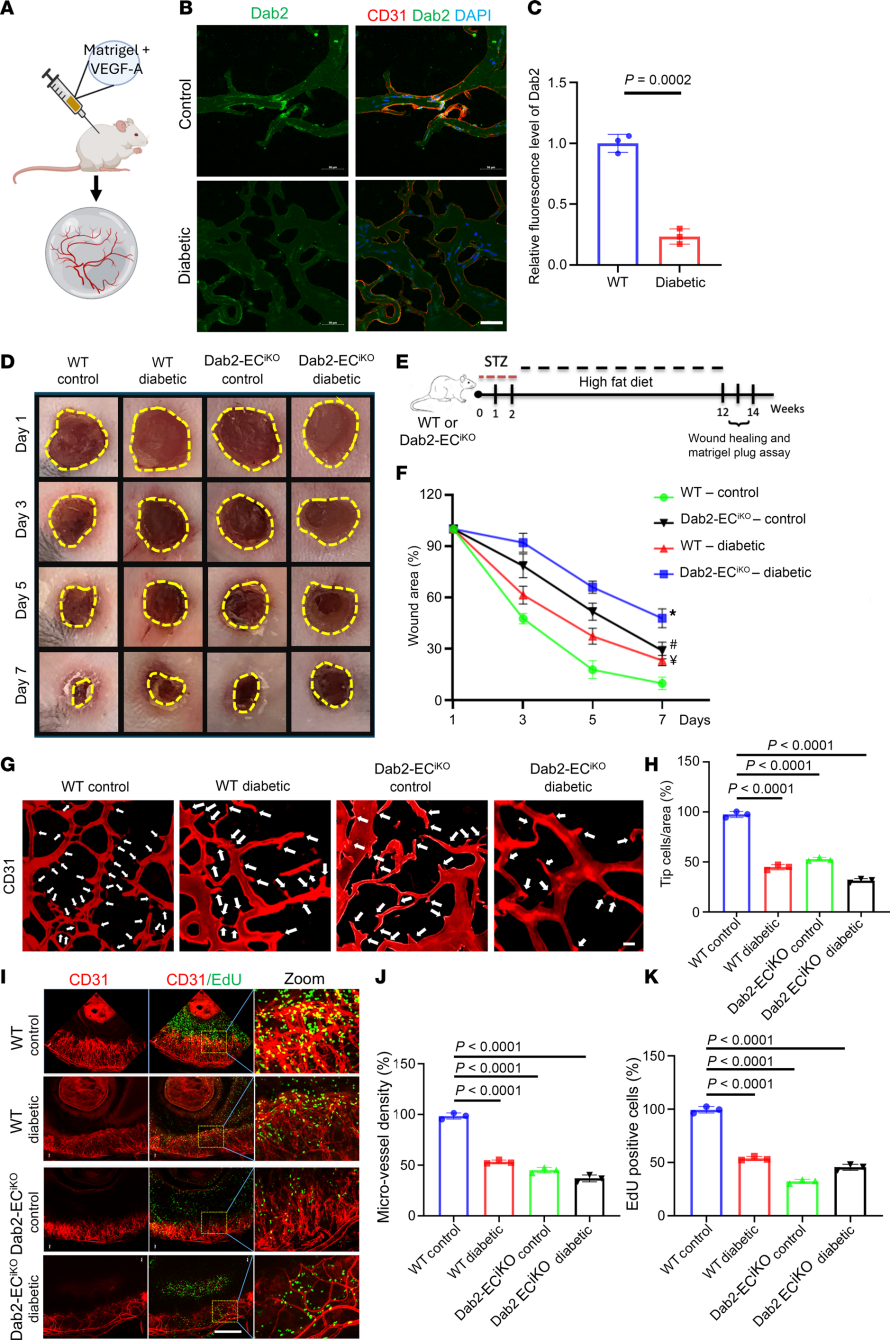
EC-specific Dab2 knockout causes reduced angiogenesis in vivo. (**A**) Schematic diagram of the Matrigel plug assay. (**B**) Representative immunofluorescence staining of Dab2 (green) and CD31 (red) in blood vessels in sections of Matrigel implant from WT and diabetic mice 1 week after injection. Scale bar = 50 μm. (**C**) Quantitation of Dab2 fluorescence intensity in **B** (*n* = 3 per group of mice, results are presented as mean ± SD, *P* value calculated by *t* test). (**D**) Representative figures of wounds from wound-healing assays in WT control mice, WT diabetes mice, Dab2-EC^iKO^ control mice, and Dab2-EC^iKO^ diabetes mice. (**E**) Schematic diagram of the protocol used to induce diabetes in mice for the wound-healing assay that illustrates the step-by-step treatment process, starting with the administration of STZ followed by an HFD regimen. (**F**) Analysis of wound closure conducted at 1, 3, 5, and 7 days after the initial wound creation, providing a timeline view of the healing process (*P < 0.05 vs. WT mice; ^#^*P* < 0.05 vs. WT mice; ^¥^*P* < 0.05 vs. WT mice. *n* = 6 per group of mice, results are presented as mean ± SD, *P* value calculated by ANOVA). (**G**) Representative immunofluorescence staining of cryosections of Matrigel implants. Scale bar = 50 μm. (**H**) Quantitation of CD31-positive tip cell percentage in **F** (*n* = 3 per group of mice, results are presented as mean ± SD, *P* value calculated by ANOVA). (**I**) Representative immunofluorescence staining of retinal micropocket assay to assess the angiogenesis. Scale bar = 500 μm. Insets were optically enlarged 4×. (**J**) Quantification of the density of blood vessels (*n* = 3 per group of mice, results are presented as mean ± SD, *P* value calculated by ANOVA). (**K**) Quantification of the density of EdU-positive proliferative skin ECs (*n* = 3 per group of mice, results are presented as mean ± SD, *P* value calculated by ANOVA).

To test Dab2 function in diabetic mice, we utilized a Matrigel transplantation technique where VEGFA-infused Matrigel was implanted into both wild-type (WT) and diabetic mice to create a conducive setting for in vivo angiogenesis analysis. This approach revealed that in diabetic mice, the angiogenic blood vessels within the VEGFA-infused Matrigel exhibited a notable decrease in Dab2 levels compared with those in the WT mice, indicating the impact of diabetes on Dab2 expression and its potential role in angiogenesis (Figure 2, A–C). To determine if the pro-angiogenic effects of Dab2 were associated with enhanced wound-healing responses in vivo, we examined the effects of EC-specific Dab2 deficiency on wound healing under physiological and diabetic conditions (Figure 2D). Mice were treated with streptozotocin (STZ) and fed a high-fat diet (HFD) to prepare the diabetic mouse model. Diabetes was induced by an intraperitoneal (i.p.) injection of STZ, with low-dose insulin given subcutaneously (s.c.) to manage mortality (19). STZ selectively damages insulin-producing β cells in the pancreas, leading to reduced insulin secretion and hyperglycemia. When used alongside HFD, which induces insulin resistance by causing obesity, this method effectively simulates the metabolic conditions of type 2 diabetes, eliciting both insulin resistance and compromised insulin production, thereby creating a comprehensive diabetic mouse model.

Glucose and insulin tolerance tests (GTTs and ITTs) were performed in WT mice and EC-specific Dab2-knockout (Dab2-EC^iKO^) mice with or without STZ injection and HFD feeding. It was found that EC-specific Dab2 deficiency led to more severe insulin resistance and higher blood glucose compared with WT mice (Supplemental Figure 2, A and B). Wounds were inflicted in the dorsal skin of normal or diabetic WT or Dab2-EC^iKO^ mice with a disposable biopsy punch under sterile conditions. Each wound was photographed at the indicated times and analyzed. EC-specific Dab2 deficiency was consistently associated with delayed wound-healing response compared with WT mice (Figure 2, D–F). Whereas diabetic conditions hampered wound healing in WT mice, Dab2-EC^iKO^ diabetic mice displayed the slowest wound-healing rate among all the groups. Consistently, CD31-specific immunofluorescence staining of wounds isolated on day 7 after wound creation revealed significantly less vascularization of the wound area in diabetic and Dab2-EC^iKO^ mice (Supplemental Figure 2, C and D).

To evaluate the effect of Dab2 on neovascularization in vivo, we subcutaneously implanted Matrigel plugs containing VEGFA into Dab2-EC^iKO^ and WT adult diabetic or control mice to directly examine EC migration and network formation in vivo. Consistently, the diabetic and Dab2-EC^iKO^ groups showed significantly reduced vascularization compared with WT controls (Figure 2, G and H). Furthermore, Matrigel from diabetic WT mice exhibited reduced vascularization to a similar extent to that in Dab2-EC^iKO^ mice.

To further investigate the impact of EC-specific Dab2 deficiency on angiogenesis, we performed a neo-angiogenesis assay induced by exogenous supplementation of VEGFA in the cornea using a corneal micropocket assay. Immunofluorescence staining of whole-mount corneas with a CD31-specific antibody verified the impaired vascularization and revealed reduced vessel density in Dab2-EC^iKO^ and diabetic mice (Figure 2, I and J). Specifically, we observed a significant decrease in the number of 5-ethynyl-2′-deoxyuridine–positive (EdU-positive) cells in the cornea of diabetic and Dab2-EC^iKO^ mice, indicative of a reduction in the proliferative response of ECs to VEGFA stimulation (Figure 2K). Taken together, these observations demonstrate that EC Dab2 plays a significant role in promoting VEGFA-driven angiogenesis and wound healing in vivo.

### Dab2 downregulation in ECs causes reduced angiogenesis in vitro.

**Figure 3:**
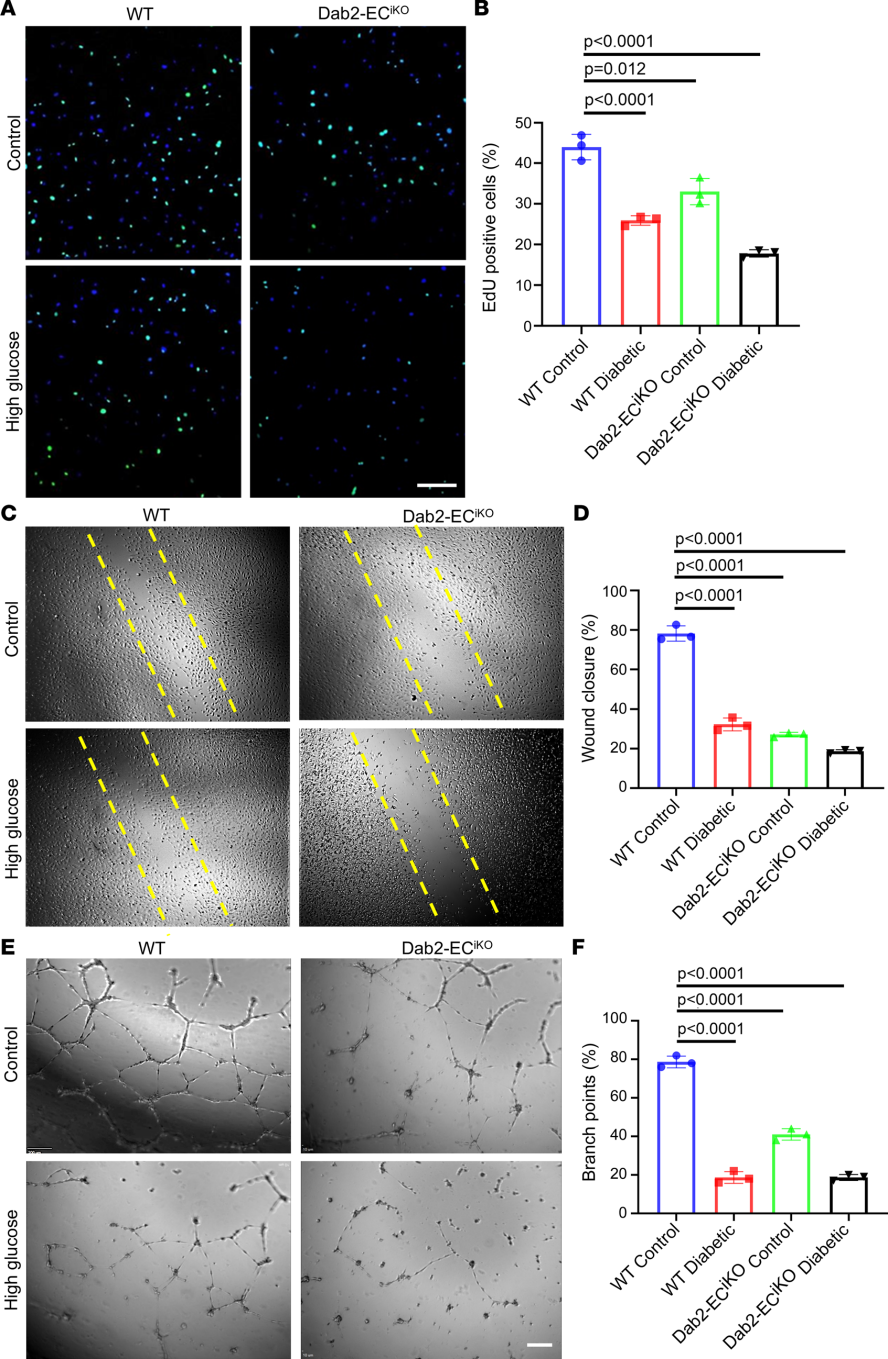
EC-specific Dab2 knockout causes reduced angiogenesis in vitro. (**A**) Representative figures of EdU incorporation (green) in WT skin ECs cultured in normal or high concentration of glucose and skin ECs from Dab2-EC^iKO^ mice with or without high concentration of glucose. Scale bar = 200 μm. (**B**) Quantitation of the proportion of EdU-positive skin ECs in **A** (*n* = 3 cell repetitions, results are presented as mean ± SD, *P* value calculated by ANOVA). (**C**) Representative figures of wound closure scratch assay of ECs monolayers as described in **A**. Original magnification, 10×. (**D**) Quantitation of wound closure results in **C** (*n* = 3 cell repetitions, results are presented as mean ± SD, *P* value calculated by ANOVA). (**E**) Representative figures of tube formation assay of cells as described in **A**. (**F**) Quantitation of branch points in results from **E** (*n* = 3 cell repetitions, results are presented as mean ± SD, *P* value calculated by ANOVA).

To further investigate the role of Dab2 in angiogenesis, we isolated skin ECs from WT and Dab2-EC^iKO^ mice and treated them with tamoxifen. We performed in vitro proliferation by EdU labeling, scratch wound healing, and EC tube formation in Matrigel in normal or high-glucose medium in the presence of VEGFA. The EdU-positive cells were significantly decreased in Dab2-deficient ECs compared with WT controls. Moreover, high-glucose treatment led to a reduced number of EdU-positive cells in WT ECs compared with culture in the medium with normal glucose concentration (Figure 3, A and B). Likewise, the in vitro scratch wound–healing assay demonstrated that Dab2-deficient ECs exhibited a slower rate of wound closure compared with WT ECs. High-concentration glucose treatment of WT ECs led to blunted scratch wound closure like ECs from Dab2-EC^iKO^ mice (Figure 3, C and D). Similarly, the in vitro tube formation assay revealed that Dab2-deficient ECs formed fewer and less organized networks in the presence of VEGFA compared with WT ECs (Figure 3, E and F). These findings suggest that Dab2 plays a crucial role in regulating the proliferation and migration of skin ECs under both normal and diabetic conditions. Inhibition of Dab2 expression likely underlies the compromised angiogenic function in ECs exposed to hyperglycemia in diabetes mellitus.

**Figure 4:**
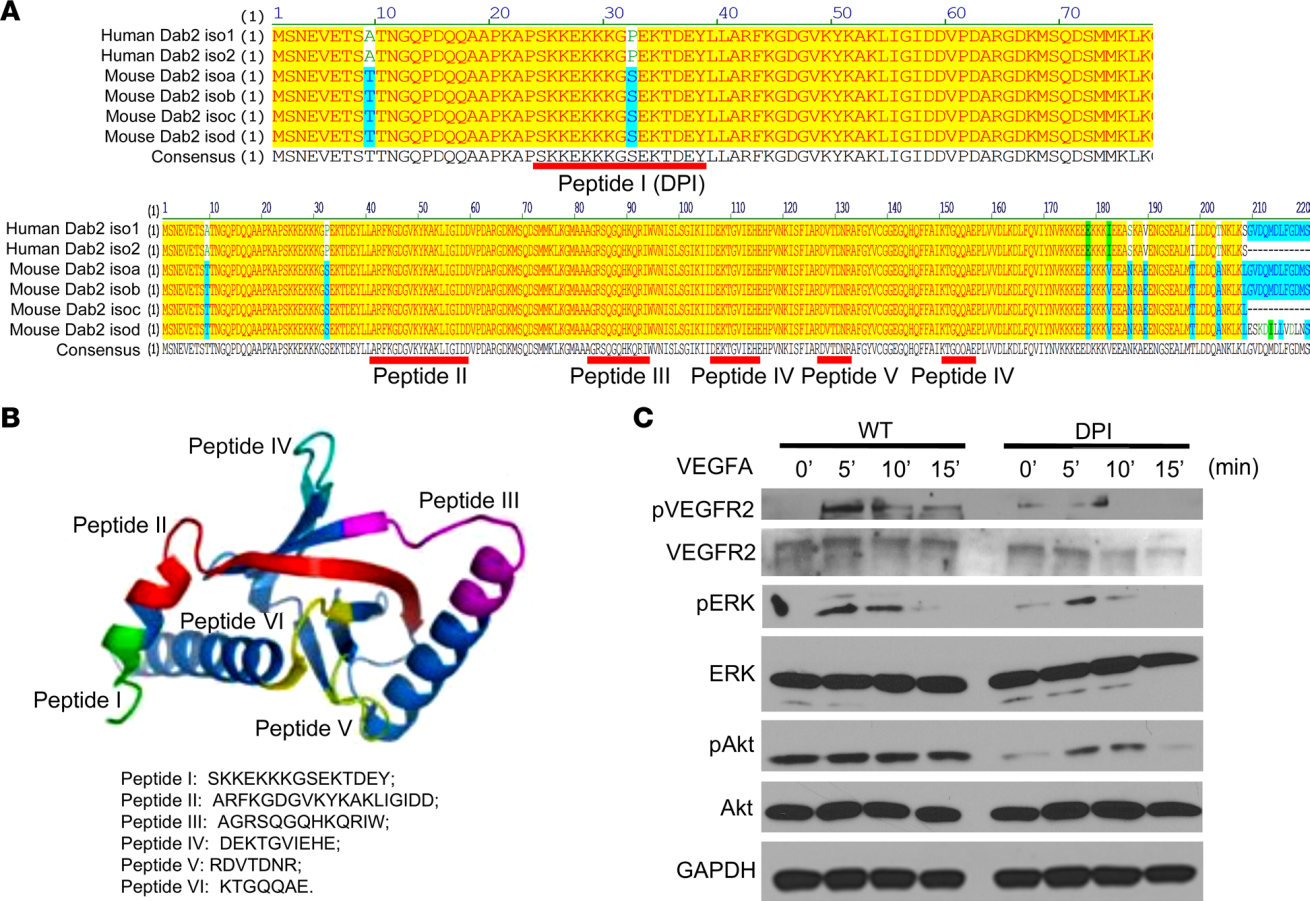
Dab2 integrin binding sites and effect of DPI on VEGFR2 signaling in skin ECs. (**A**) Alignment of human and mouse Dab1 and Dab2 protein-coding sequences, identifying a consistent RGD motif and an additional KGD motif in the Dab2 PTB domain, suggesting evolutionarily conserved integrin binding capabilities. (**B**) The presence of an RGD peptide motif and an additional KGD motif in the Dab2 PTB domain suggests integrin binding capabilities. (**C**) Immunoblot of VEGFR2-proximal signaling components in skin ECs pretreated with DPI followed by VEGFA stimulation.

Dab2 is known to affect various cellular processes and is a critical regulator of VEGFR2 signaling. A previous study has shown that Dab2 could affect the VEGFR2 signaling pathway in glomerular ECs (13). However, the binding domain of *Dab2* with VEGFR2 is not clear. To determine whether Dab2 could activate VEGFR2 signaling in angiogenesis and determine the precise binding domain involved in this activation, we next sought to determine exogenous inhibition of Dab2 with an inhibitory peptide (DPI) that could impede VEGFR2 signaling. We predicted a minimal peptide stretch in the Dab2 phosphotyrosine-binding (PTB) domain, which is predicted to associate with VEGFR2, would abolish the interaction between VEGFR2 and Dab2 under various conditions. Using structural bioinformatics and molecular modeling, the study analyzed the alignment of human and mouse Dab2, identifying a consistent arginyl-glycyl-aspartic acid (RGD) motif and an additional lysine-glycine-aspartic acid (KGD) motif in the Dab2 PTB domain, suggesting integrin binding capabilities (Figure 4, A and B). WT mouse ECs were pretreated with DPI or control peptides for 18 hours, followed by VEGFA stimulation. Consistent with the observations made in Dab2-depleted ECs, the DPI peptide substantially reduced the levels of phosphorylated (p-) VEGFR2, p-AKT, and p-ERK relative to the control peptide (Figure 4C). Together, these results suggest that Dab2 plays a crucial role in regulating the VEGF/VEGFR2 signaling pathway in EC angiogenesis.

### Restoration of Dab2 expression in ECs rescues impaired angiogenesis and wound healing in diabetic mice.

**Figure 5:**
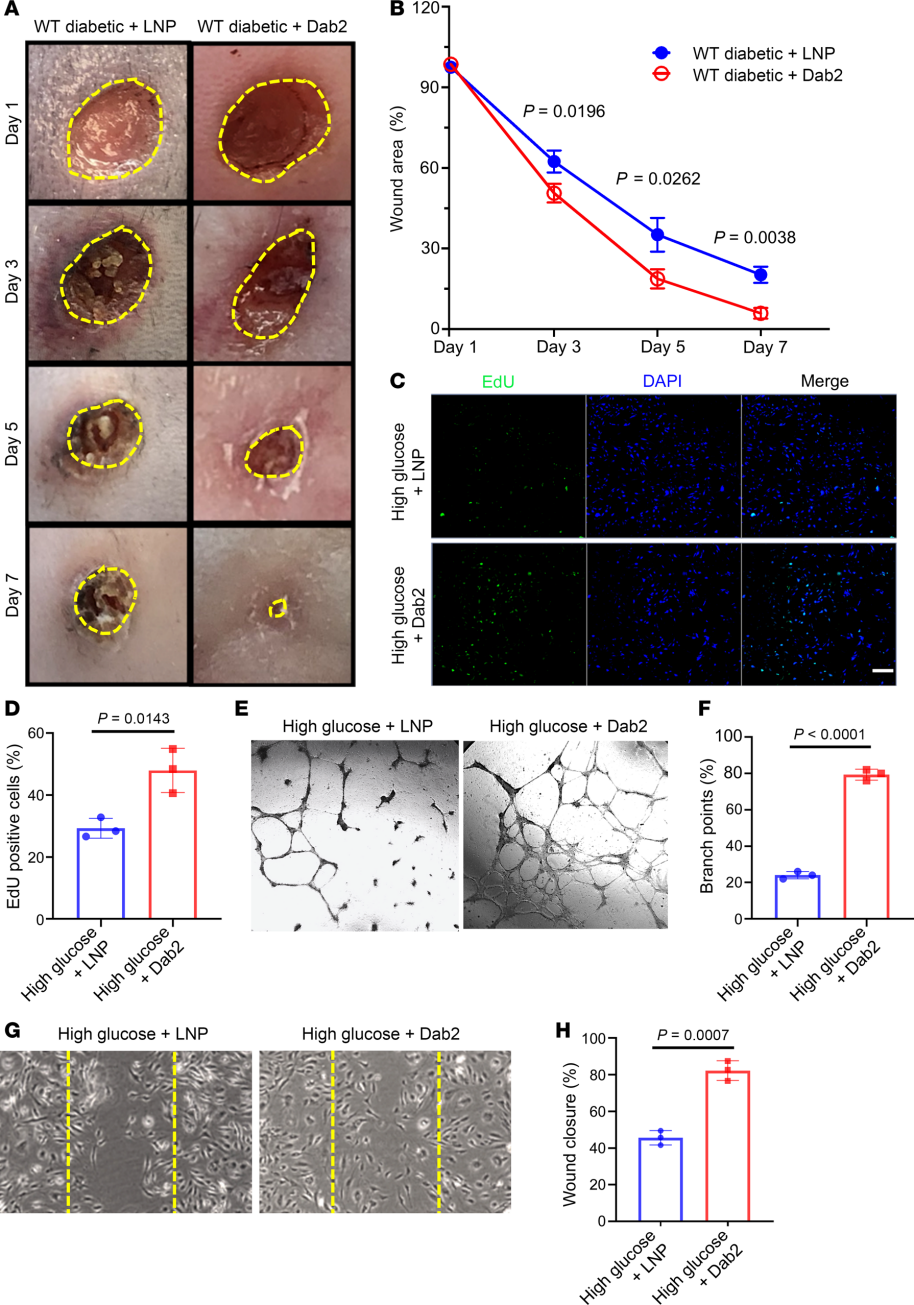
Restoration of Dab2 expression in ECs rescues impaired angiogenesis and wound healing in diabetic mice. (**A**) Representative figures of wounds from wound-healing assays in WT diabetic mice treated with LNPs carrying mRNAs encoding GFP (WT diabetic + LNP group) or *Dab2* (WT diabetic + Dab2 group). (**B**) Quantitation of wound closure conducted on days 1, 3, 5, and 7 after the initial wound (*n* = 5 per group of mice, *P* value calculated using *t* test). (**C**) Representative figures of EdU of skin ECs cultured in high concentration of glucose infected with empty lentivirus vector or lentivirus carrying *Dab2* cDNA. Scale bar = 200 μm. (**D**) Quantitation of the proportion of EdU-positive cells in **C** (*n* = 3 cell repetitions, *P* value calculated using *t* test). (**E**) Representative figures of tube formation assay on skin ECs cultured in high concentration of glucose and infection with empty vector or lentivirus carrying *Dab2* cDNA. Original magnification, 100×. (**F**) Quantitation of branch points in results from **E** (*n* = 3 cell repetitions, results are presented as mean ± SD, *P* value calculated by *t* test). (**G**) Representative figures of wound-healing assay on skin ECs cultured in high concentration of glucose and infection with empty vector or lentivirus carrying *Dab2* cDNA. Original magnification, 100×. (**H**) Quantitation of wound closure results in **G** (*n* = 3 cell repetitions, results are presented as mean ± SD, *P* value calculated by *t* test).

To investigate the therapeutic efficacy of Dab2 restoration in diabetic conditions, we conducted a rescue experiment in STZ- and HFD-induced diabetic mice. We intravenously (i.v.) administered *Dab2* mRNA encapsulated in LNPs conjugated with LyP-1 peptide at a dose of 15 μg/mouse, twice a week, during the wound-healing period (Supplemental Figure 3). This treatment was aimed at restoring Dab2 expression and enhancing angiogenesis and wound healing. Diabetic mice treated with LNPs-LyP-1-*Dab2* mRNA exhibited significantly accelerated wound-healing activity compared with the untreated diabetic mice (Figure 5, A and B). The treated group exhibited accelerated wound closure and healing rates, with most wounds nearly fully healed by day 7, in contrast with the control group (LNPs-LyP-1-GFP mRNA), where wound healing was significantly impeded within the same time. To complement these in vivo findings, we utilized a *Dab2* lentivirus system to overexpress Dab2 in high-glucose-treated primary skin ECs enriched from adult mice. Dab2 overexpression in high-glucose treatment recovered the proliferative capacity in ECs compared with control (Figure 5, C and D). We also performed scratch assays and tube formation assays to mimic wound healing and angiogenesis in vitro (Figure 5, E–H). Cells overexpressing Dab2 tended to form more organized and complex tube-like structures, as revealed by tube formation assays (Figure 5, E and F), and exhibited increased migratory behavior in scratch assays (Figure 5, G and H), compared with control cells. These results verify the EC-autonomous, pro-angiogenic function of Dab2 and highlight the potential therapeutic utility of overexpressing Dab2 to restore impaired angiogenic responses in diabetic conditions.

### FOXM1 is downregulated in diabetes and regulates Dab2 transcription.

**Figure 6:**
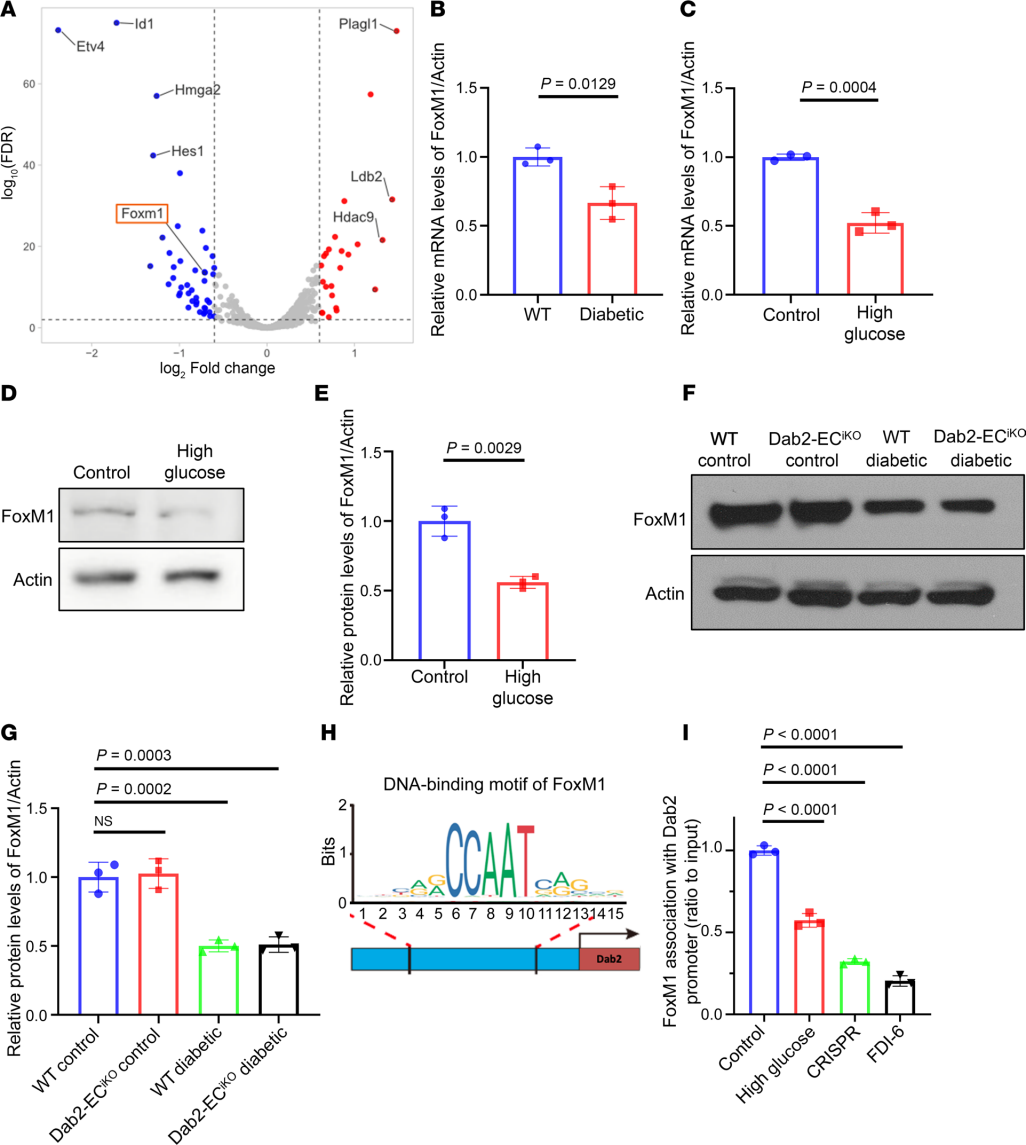
FOXM1 is downregulated in diabetes and regulates *Dab2* transcription. (**A**) Volcano plot of differentially expressed transcription factors in skin ECs cultured in high versus normal concentration of glucose for 48 hours. The *x* axis shows the log_2_FC, and the *y* axis represents the –log_10_ FDR (*n* = 3 per group of mice). (**B**) RNA abundance of *Foxm1* in ECs isolated from normal or diabetic mouse skin determined by qRT-PCR (*n* = 3 cell repetitions, results are presented as mean ± SD, *P* value calculated by *t* test). (**C**) RNA abundance of *Foxm1* in skin ECs cultured in normal or high concentration of glucose determined by qRT-PCR (*n* = 3 cell repetitions, results are presented as mean ± SD, *P* value calculated by *t* test). (**D**) Representative Western blots of Dab2 in skin ECs cultured in control or high concentration of glucose. (**E**) Quantitation of protein level of Dab2 relative to Actin in **D** (*n* = 3 cell repetitions, results are presented as mean ± SD, *P* value calculated by *t* test). (**F**) Representative Western blots of Dab2 in skin ECs isolated from WT control mice, Dab2-EC^iKO^ control mice, WT diabetic mice, and Dab2-EC^iKO^ diabetic mice. (**G**) Quantitation of protein level of Dab2 relative to Actin in **E** (*n* = 3 cell repetitions, results are presented as mean ± SD, *P* value calculated by *t* test). (**H**) JASPAR-predicted FOXM1-binding site in the *Dab2* promoter. (**I**) FOXM1 binding to the *Dab2* promoter in ECs exposed to high concentration of glucose, or FDI-6, or with a CRISPR-mediated deletion mutation in the FOXM1-binding site on the *Dab2* promoter (*n* = 3 cell repetitions, results are presented as mean ± SD, *P* value calculated by ANOVA).

To find the mechanism of *Dab2* regulation, we further explored the RNA-sequencing data in Figure 1. Volcano plot analysis revealed downregulation of transcription factors involved in cell proliferation and growth, including FOXM1, early growth response protein 2 (Egr2), hairy and enhancer of split 1 (Hes1), and ETS variant 4 (Etv4), exclusively in the high-glucose-treated samples compared with the control group (Figure 6A and Supplemental Figure 1). qPCR analysis showed that high-glucose treatment of ECs from diabetic mice resulted in the downregulation of Dab2 (Figure 6, B and C). Similar to the qPCR results, Western blot analysis revealed significantly downregulated FOXM1 protein levels in the diabetic group or high-glucose-treated group compared with nondiabetic controls (Figure 6, D–G).

The concomitant downregulation of *FoxM1* and *Dab2* transcripts under diabetic conditions raises the possibility that, as a regulatory transcription factor, FOXM1 directly influences Dab2 expression to modulate VEGFR2-mediated endothelial function. FOXM1 has been shown to promote cell cycle progression, cell proliferation, and cellular metabolism (20, 21), while Dab2 is a critical regulator of VEGFR2 signaling. To investigate how FOXM1 regulates *Dab2* expression and VEGFR2 signaling, we used chromatin immunoprecipitation–qPCR (ChIP-qPCR) analysis in ECs to determine if FOXM1 can bind to the *Dab2* promoter and other regulatory regions. We isolated ECs from WT mice. We used the JASPAR database (22, 23) to predict potential FOXM1 transcription factor binding sites. A potential binding site within the promoter region of the *Dab2* locus with the highest score was selected for further study (Figure 6H and Supplemental Figure 4A). ChIP-qPCR results showed that indeed FOXM1 bound to this predicted region within the promoter region of *Dab2* in ECs. Interestingly, the binding of FOXM1 to the *Dab2* promoter region was decreased when the ECs were treated with high-concentration glucose or the FOXM1 inhibitor FDI-6 (Figure 6I). More importantly, disrupting the FOXM1 binding site with CRISPR/Cas-induced mutation in ECs also significantly diminished FOXM1 binding.

### FOXM1 inhibitor FDI-6 downregulates Dab2 expression and the phosphorylation of VEGFA-induced VEGFR2.

The significance of FOXM1 in ECs and vascular repair is underscored by its role in promoting endothelial regeneration and resolving inflammatory lung injury. *FoxM1*, expressed during embryogenesis in various cell types, including ECs, is crucial for pulmonary vascular ECs’ proliferation and endothelial barrier recovery after inflammatory injury. Notably, FOXM1 facilitates the reannealing of endothelial adherens junctions, enhancing endothelial barrier function after vascular injury. Aging-related impairment in endothelial regeneration and inflammatory injury resolution is linked to inadequate FOXM1 induction, which, when addressed through transgenic expression, improves outcomes in aged mice, highlighting the potential of FOXM1 as a target for vascular repair interventions (24–27).

**Figure 7:**
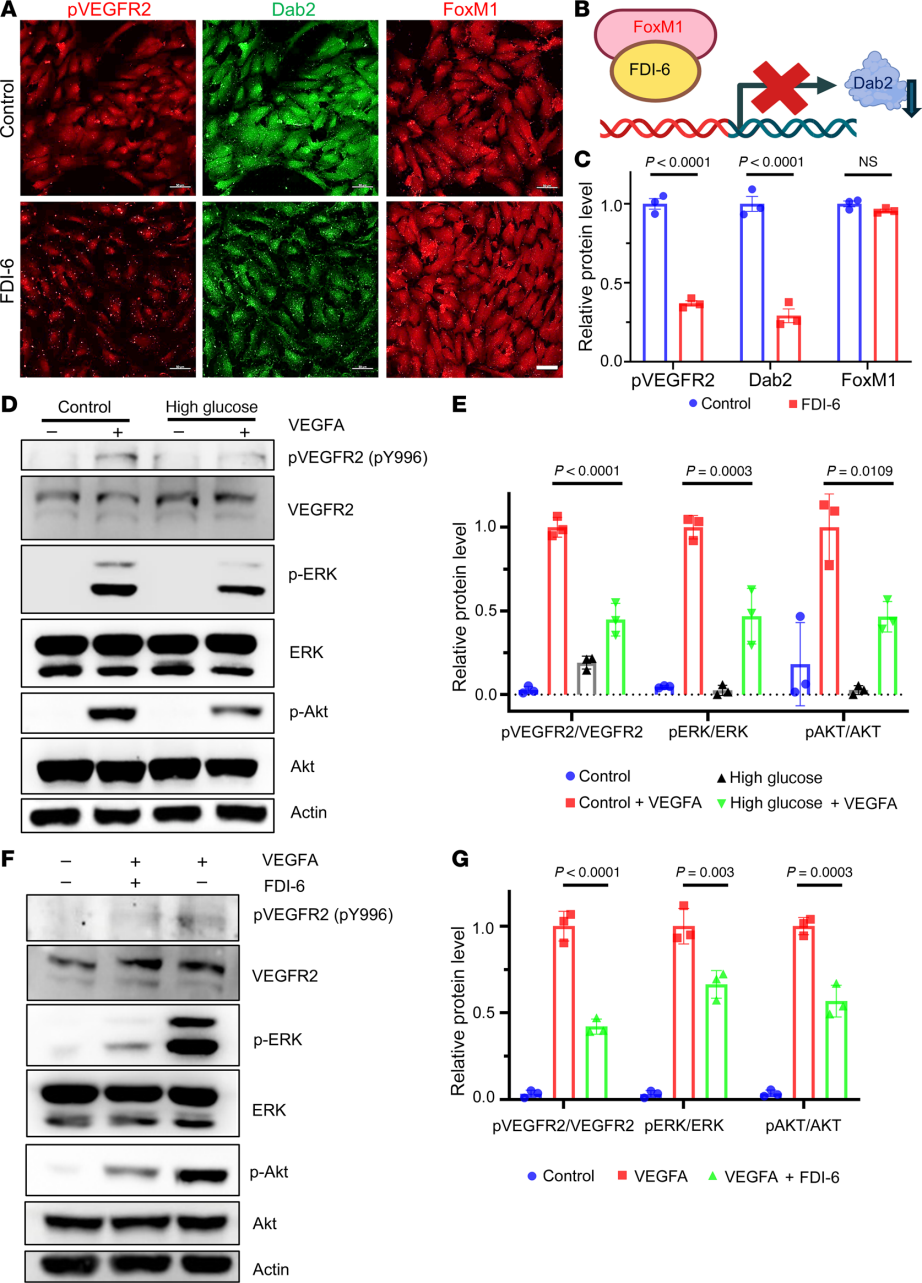
FOXM1 inhibitor FDI-6 downregulates *Dab2* expression and the phosphorylation of VEGFA-induced VEGFR2. (**A**) Representative immunofluorescence staining of skin ECs treated with or without FDI-6. Scale bars = 50 μm. (**B**) Schematic diagram showing inhibition of Dab2 expression by FDI-6. (**C**) Quantitation of the immunofluorescence intensity in **A** (*n* = 3 cell repetitions, results are presented as mean ± SD, *P* value calculated by *t* test). (**D**) VEGFA-induced phosphorylation of key VEGFR2-proximal signaling components in skin ECs treated in control or high-glucose concentration with or without VEGFA assessed by Western blot analysis. (**E**) Quantitation of results described in **D** (*n* = 3 cell repetitions, results are presented as mean ± SD, *P* value calculated by ANOVA). (**F**) Representative Western blot of VEGFA-induced key VEGFA-induced VEGFR2-proximal signaling in skin ECs treated with or without FDI-6. (**G**) Quantitative analysis of immunoblots in **F** (*n* = 3 cell repetitions, results are presented as mean ± SD, *P* value calculated by ANOVA).

To determine if FOXM1 controls *Dab2* transcription, we used the small molecule FDI-6 to specifically inhibit FOXM1 function. FDI-6 is a FOXM1 transactivational inhibitor that blocks its DNA binding (28, 29). Immunofluorescence (IF) staining of primary ECs revealed a significant decrease in the expression of p-VEGFR2 and Dab2 in FDI-6–treated conditions compared with the control group, while the expression of FOXM1 remained unchanged (Figure 7, A–C). In contrast, the total protein level of FOXM1 was unaffected by FDI-6 treatment. In the VEGF signaling pathway, ERK and AKT would normally be phosphorylated and activated. After high-glucose treatments, we observed diminished phosphorylation and activation of VEGFR2 (p-VEGFR2), AKT (p-AKT), and ERK (p-ERK) (Figure 7, D and E). Furthermore, FDI-6 treatment reduced the protein levels of p-VEGFR2 as well as activation of both p-ERK and p-AKT (Figure 7, F and G). These observations suggest that FOXM1 regulates the expression of Dab2 which, in turn, controls VEGFR2 signaling.

## Discussion

Chronic nonhealing wounds in diabetes present a complex challenge, and impaired angiogenesis appears to underpin these defects in tissue repair and regeneration. Blunted angiogenesis is often attributed to dysregulated signaling and aberrant gene expression precipitated by diabetic conditions. Therefore, identifying crucial angiogenesis modulators is crucial to illustrate the pathogenesis of blunted angiogenesis in diabetes and assess their therapeutic utility. Our observations here provide important insights into the precise involvement of the highly conserved endocytic adaptor protein, Dab2, and its transcriptional regulation during wound-induced angiogenesis in diabetes. Dab2 is widely expressed in various tissues, suggesting tissue-specific and context-dependent roles in multiple physiological processes (30). Dab2 primarily functions as a cytosolic, clathrin and cargo binding adaptor protein, with a pivotal role in endocytosis. By facilitating the internalization of cargo molecules and the binding of clathrin-coated vesicles to cargo proteins through clathrin-mediated endocytosis, Dab2 not only facilitates signal transduction and receptor recycling but also plays a crucial role in maintaining cellular homeostasis and regulating intracellular trafficking (31, 32).

Intriguingly, Dab2 and its phosphorylated form are enriched in ECs (13, 33). Studies in *Xenopus* and *Zebrafish* have revealed a functional contribution of Dab2 to developmental angiogenesis through both VEGF-dependent and VEGF-independent mechanisms (34–37). Earlier in vitro studies revealed that Dab2 function is conducive to EC migration via mitigating VEGF signaling (30, 38). Similarly, Dab2 is required for the vascularization of the brain tissue and the establishment of the neurovascular unit in part by enhancing VEGF signaling (39, 40). At least 2 other studies suggested that activated receptor endocytosis through Dab2 results in augmented VEGF signaling in ECs (14, 37). Nakayama et al. showed that phosphorylation of its PTB domain diminishes its interaction with the VEGF pathway receptors, VEGFR2 and VEGFR3, revealing the specificity of Dab2 for VEGFRs (14). However, the upstream regulatory mechanisms moderating the expression of Dab2 and its interaction with VEGFR2 remain unknown. Furthermore, despite the well-established pro-angiogenic role of Dab2, the therapeutic potential of exploiting Dab2-mediated angiogenesis to enhance wound healing in a disease context (i.e., diabetic wounds), has remained unexplored.

In our quest to investigate the involvement of endocytic adaptor proteins in mitigating aspects of diabetes, we identified Dab2 via our bulk RNA-sequencing analysis of hyperglycemic ECs as one of the downregulated genes. Similarly, we observed that ECs isolated from the STZ-induced diabetic mice model showed a significant downregulation of *Dab2* mRNA and protein levels. Although the precise involvement of Dab2 in diabetes remains to be addressed, Dab2 appears to be involved in regulating blood glucose metabolism; its deficiency, at least in myeloid cells, has been implicated in compromised glucose tolerance in mice (41). More intriguingly, and only recently, polymorphisms in the *Dab2* gene have been associated with type 2 diabetes mellitus in a population-based study (36).

Because of the lack of information about the precise role of Dab2 in diabetes, we went on to develop an EC-specific Dab2-deficient mouse model to examine the effects of Dab2 deletion on angiogenesis in diabetic conditions. We observed that EC-specific loss of Dab2 was strongly associated with delayed wound-healing response, and a diabetic background further exacerbated this process in Dab2-EC^iKO^ mice, coupled with severely blunted angiogenesis. Meanwhile our in vitro model revealed that loss of endothelial Dab2 resulted in curtailed angiogenesis, as evidenced by diminished cell migration, network formation, and proliferation.

Mechanistically, Dab2 deletion affected VEGFR2 activation, thereby affecting vascular development both in vitro and in vivo. The DPI peptide, which specifically blocked Dab2 interactions with VEGFR2, could downregulate VEGF2 activation in VEGFA-stimulated ECs. These results align with prior studies on the role of Dab2 in angiogenesis but introduce insights, including a comprehensive in vivo evaluation of angiogenesis in the diabetic context. This unique approach enhances our understanding in dissecting the pathological mechanisms of Dab2 underlying diabetic vascular complications.

Type 2 diabetes has many complications, including diabetic kidney disease, diabetic retinopathy, diabetic neuropathy, and delayed and impaired wound healing. The delayed wound healing in diabetes brings about complications, such as foot ulcer and even lower extremity amputations. Ulceration at a lower limb could cause deep tissue infections, foot deformities, and peripheral arterial disease. Now, the therapy of foot ulcer is based on glycemic control and debridement, including autolytic, enzymatic, and biological. Immunomodulators like honey and nitric oxide, growth factors, inhibitors of metalloproteinases, autologous platelet-derived gels, bioengineered skin substitutes, and oxygen therapy are currently used as therapy (3). Meanwhile, new foot ulcer therapies are under investigation in ongoing clinical trials, which are mainly focusing on exploring drug delivery. Nanoparticle/nanomaterials-based diabetic wound healing is highlighted because it is more bioavailable and quickens wound healing. Three-dimensional polymer hydrogels (42), nanofibers (43), and nonpolymeric nanoparticles including silver nanoparticles and gold nanoparticles are all used in foot ulcer therapy (44). Li et al. showed that VEGF circular RNA NPs could improve wound healing in diabetic mice (45). This novel therapeutic approach bypasses common encumbrances associated with other modes of delivery, such as inadequate absorption or shorter half-life of delivered proteins. Consistently, a recent study has demonstrated the safety and specificity of augmenting VEGFA via nanoparticles (46). The inhibitory effect of EC-specific Dab2 knockout on wound healing in our results suggested that exogenous supplementation of Dab2 could be a therapeutic approach. Consequently, we investigated the effects of exogenous Dab2 restoration on angiogenesis in vivo and in vitro. In this study, we employed a potentially novel method to administer Dab2 supplementation via the deployment of *Dab2* mRNA encapsulated in LNPs conjugated with LyP-1 peptide. The LNPs-LyP-1-*Dab2* mRNA group exhibited significantly accelerated wound-healing activity compared with the untreated diabetic siblings, as observed by enhanced wound closure and healing rates, with most wounds fully healed by day 7 (Figure 5, A and B). This result demonstrated the feasibility and positive effect of *Dab2* mRNA supplementation in vivo. However, more in vivo experiments are needed to fully explore the dosage and usage of mRNA nanoparticles in this context. Problems such as drug degradation and ineffective drug release also need to be solved. Therefore, *Dab2* mRNA nanoparticle therapy has good prospects and needs more research focusing on it. The beneficial effects of Dab2 restoration on angiogenesis also suggested that supplementation of Dab2 may serve as a therapeutic target for diabetic delayed wound healing.

Further delving into the mechanism, we demonstrated that FOXM1, a highly conserved transcription factor, exerts regulatory control over *Dab2* expression in ECs during angiogenesis associated with wound healing. Notably, we showed that FOXM1 binds to the *Dab2* promoter to drive *Dab2* expression. Therefore, we have established that FOXM1 exerts a positive regulatory control on *Dab2* transcription, adding further insights into the molecular mechanisms regulating angiogenesis during wound healing in diabetic conditions.

Although previous studies have suggested an involvement of FOXM1 in the pathogenesis of diabetes, emerging studies have revealed that deletion of FOXM1 in diabetic mice impairs wound healing, in part, via impeded recruitment of immune cells (47). However, the EC-specific role of FOXM1 in diabetic wounds was not addressed. FOXM1 plays a crucial role in β cell proliferation, essential for pancreatic repair and insulin secretion (48). It activates pathways critical for β cell growth and interacts with regulatory genes, enhancing its transcriptional activity via the insulin receptor-mediated pathway. Additionally, FOXM1’s involvement extends to nutrition-induced β cell growth and gestational diabetes, highlighting its importance in β cell function in various physiological conditions (46, 48). Although previous studies acknowledge the pivotal role of FOXM1 in diabetes-related cellular functions and β cell proliferation, our study delves into the mechanistic interaction between FOXM1 and Dab2 within the context of ECs and angiogenesis during diabetic wound healing.

Our study provides crucial insights into the role of Dab2 and FOXM1 in diabetic wound healing, highlighting the therapeutic potential of *Dab2* mRNA encapsulated in LNPs. This approach not only advances our understanding of the molecular mechanisms underlying diabetes-related angiogenesis and wound repair but also opens avenues for developing targeted treatments for diabetic complications.

## Methods

### Sex as a biological variable.

Both male and female mice were equally included in this study.

### Mouse models.

To produce Dab2-EC^iKO^ mice, a breeding strategy was employed using Dab2^fl/fl^ mice (The Jackson Laboratory, strain: 022837) and EC-specific Cre transgenic CDH5-Cre mice (The Jackson Laboratory, MGI: 3848982). To activate Cre recombinase, 8- to 10-week-old mice received 4-hydroxytamoxifen (Hello Bio, dissolved in a 9:1 mixture of dimethyl sulfoxide and ethanol at a dosage of 5–10 mg/kg body weight) 7 times every other day. For induction of diabetes, mice underwent i.p. injection with a low dose of STZ (MilliporeSigma, 50 mg/kg) following an established protocol (49). Hyperglycemia was confirmed when mice maintained a fasting blood glucose level above 200 mg/dL for over a week after STZ administration. After diabetes induction, the mice were placed on an HFD (60% kcal fat from Research Diets Inc.).

### Cell cultures.

Primary mouse ECs were obtained from mouse skin and cultured according to previously established protocols with some modifications (49–51). Briefly, to isolate ECs from the skin, 4–6 mice aged 2–3 months were used. The mice were anesthetized with isoflurane and humanely euthanized by cervical dislocation. The skin was excised from the mice using surgical scissors or a scalpel, then diced into small fragments on ice and subjected to enzymatic digestion with collagenase type IV (2 mg/mL; Gibco Laboratories) in a 37°C water bath with agitation (10 rpm) for a duration of 60–90 minutes. Digestion was stopped by adding an equal volume of ice-cold FBS. The resulting digested tissue was filtered through a 40 μm cell strainer (BD) to separate the cells from debris. The resulting cell suspension was then centrifuged at 400*g* for 5 minutes at 4°C. A total of 10 μL anti-mouse CD31 MicroBeads (Miltenyi Biotec) were added into about 10^7^ isolated cells in 90 μL of buffer (PBS at pH 7.2, 0.5% BSA, and 2 mM EDTA). The cell mixture was then incubated for 15 minutes at 4°C. These isolated cells were utilized for downstream experiments. The primary ECs used in all experiments were isolated and maintained 1–6 passages. Cells were treated with normal-glucose (5 mmol/L) or high-glucose (20 mmol/L) medium for 48 hours. ECs derived from WT mice or mice carrying Dab2^fl/fl^ iCDH5-ER^T2^ Cre alleles were exposed to 5 μmol/L of 4-hydroxytamoxifen (dissolved in ethanol) for 2 days at 37°C. Following treatment, cells were incubated for another 2 days without 4-hydroxytamoxifen. Confirmation of Dab2 deletion was carried out by Western blotting.

### Mouse corneal micropocket angiogenesis assay.

The corneal micropocket angiogenesis assay in mice was carried out following established protocols (49, 52). Briefly, mice were anesthetized using Avertin (400–500 mg/kg delivered by i.p. injection). An incision into the cornea was gently created at an approximately 30° angle and 0.7–1.0 mm from the limbus using a corneal blade and a stereoscope. A sustained-release pellet containing the volume of 0.4 mm × 0.4 mm × 0.2 mm pellet of VEGFA (~20 ng, BioLegend) was implanted into the pocket. At 5–7 days postimplantation, the corneas were excised and stained with PE-conjugated anti-CD31 antibody (1:100, BD Pharmingen) to highlight limbal blood vessels. The growth of these vessels was quantified by measuring the growth pixels using the Vessel Analysis plugin in ImageJ (NIH).

### RNA isolation.

Total RNA was isolated from the cells using a commercially available kit (QIAGEN) according to the manufacturer’s instructions. RNA quantity and quality were determined using a NanoDrop 2000 spectrophotometer (Thermo Fisher Scientific).

### Library preparation and bulk RNA sequencing.

Total RNA was extracted from the collected samples and assessed for quality and integrity. Quality control of all RNA-Seq samples was performed at the Harvard Medical School Biopolymer Facility using a 2100 Bioanalyzer (Agilent). Samples with an RNA integrity number score greater than 7 were considered for further processing. The NEBNext Poly(A) mRNA Magnetic Isolation Module (New England Biolabs) was used to enrich poly(A)^+^ mRNA from the total RNA pool, using Oligo d(T)25 beads for magnetic separation. After isolation, the mRNA was eluted and either used immediately or stored at –80°C for subsequent experiments. Library preparation began with reverse transcription of the enriched mRNA into cDNA, followed by end repair and the addition of a single A nucleotide at the 3′ ends. NEBNext Adaptors (New England Biolabs) were then ligated to the cDNA, and unique indices were added using the NEBNext Multiplex Oligos for Illumina to allow for sample multiplexing during sequencing. The adaptor-ligated cDNA was used for PCR amplification, and the product was purified to remove any remaining enzymes and primers. The final library was validated for quality using capillary electrophoresis and quantified through qPCR. Libraries were pooled in equimolar ratios, as determined by the quantification results, before being forwarded to the sequencing facility for high-throughput analysis. The RNA library was sequenced using the HiSeq next-generation sequencing system (Illumina) with a read length of 75 at the core facility of Azenta Life Sciences.

### Bulk RNA sequencing data analysis.

Sequencing data were processed using Trimmomatic software. Low-quality reads were removed to retain only high-quality sequences for analysis. These clean readings were then aligned to the mouse reference genome GRCm38 (mm10) using the HISAT2 alignment tool. StringTie was used to enumerate the reads for each gene. The edgeR package in R was used to identify genes with significant changes in expression, with significant differences being noted at an adjusted *P* value of less than 0.05. Pathway and gene function analyses were performed using gene set enrichment analysis, with significance attributed to terms with an adjusted *P* value and FDR below 0.05. Genes were categorized in the volcano plot based on the degree of expression change and statistical significance, with colors assigned accordingly.

### Quantitative reverse transcriptase PCR.

Total RNA was isolated from primary mouse ECs using a commercially available kit (QIAGEN) followed by DNase I treatment. The RNA was then reverse-transcribed into cDNA using Oligo (dT) 20 Primers (Invitrogen) as per the manufacturer’s protocol. Quantitative real-time PCR was performed using the StepOnePlus Real-Time PCR detection system (Applied Biosystems) and SYBR Green qPCR Supermix (Invitrogen). The PCR amplification cycles consisted of an initial heating step at 95°C for 10 minutes, followed by 40 cycles of 15 seconds at 95°C, 1 minute at 60°C, and 45 seconds at 72°C. The relative abundance of mRNA was determined using the average threshold cycles of samples normalized to β-actin mRNA. Each sample was analyzed in triplicate. The mouse primer sequences used for PCR are shown in Supplemental Table 2.

### RNA interference.

The study used RNA interference (RNAi) to knock down the expression of specific genes in primary ECs. ON-TARGETplus Mouse *Dab2* siRNA (Horizon, J-050859-09-0002) and its respective ON-TARGETplus nontargeting siRNAs (Horizon, D-001810-0X) were transfected in the isolated ECs using either Oligofectamine or RNAiMAX according to the manufacturer’s instructions (Invitrogen). The cells were processed for biochemical immunoprecipitations or immunofluorescence assays 48–72 hours after transfections as previously described (53).

### GTT.

Glucose tolerance was tested as described previously (49). Mice were fasted for 16 hours. The weight of each mouse determined the calculated glucose dose at a ratio of 2 g/kg body weight: injection volume into the peritoneal of mouse = BW (g) × 10 μL of 250 mg/mL glucose solution. Tail snipping was used to collect blood samples. Blood glucose was determined by glucometer in tail vein blood. Blood glucose was measured at 0, 30, 60, 90, and 120 minutes after glucose injection.

### ITT.

Insulin tolerance was tested as described previously (49). Mice were fasted for 3 hours. The weight of each mouse determined the calculated insulin dose at a ratio of 0.75 U/kg body weight. Insulin was prepared at 0.1 U/mL in advance (16.6 μL of 10 mg/mL insulin in 40 mL PBS): injection volume into the peritoneal of mouse = BW (g) × 7.5 μL of 0.1 U/mL insulin solution. Tail snipping was used to obtain blood, and glucose levels were determined using a glucometer. Measurements were made at 0, 15, 30, 60, 90, and 120 minutes after insulin injection.

### LyP-1 peptide–linked Dab2 and control mRNA LNPs.

LyP-1 peptide was conjugated to DSPE-PEG via an NHS-amine reaction (54–57). Briefly, the activated DSPE-PEG-NHS was mixed with LyP-1 in 1× PBS buffer solution (pH 7.4) at room temperature and stirred for 24 hours. These crude products were dialyzed against water for 3 days (MW cutoff, 3 kDa), followed by lyophilization. Successful conjugation was confirmed using proton nuclear magnetic resonance (^1^H NMR) (58).

LNPs containing mRNA, including LNPs-LyP-1-*Dab2* mRNA and LNPs-LyP-1-GFP mRNA were formulated by mixing an aqueous phase containing mRNA and an organic ethanol phase containing MC3, 1,2-dioleoyl-sn-glycero-3-phosphoethanolamine (DOPE), cholesterol, and PEG-conjugated lipids (DMG-PEG and DSPE-PEG) (59, 60). *Dab2* mRNA was synthesized from TriLink BioTechnologies by using the *Dab2* mRNA sequence NM_023118.5. Briefly, 1 volume of lipid mixtures (MC3, DOPE, Chol, DMG-PEG, and DSPE-PEG-LyP-1 at a molar ratio of 50:10:38:1:1) in ethanol and 3 volumes of mRNA (*Dab2* mRNA or GFP mRNA, 1:10 w/w mRNA to lipid) containing sodium acetate buffer (50 mM, pH 4) were mixed thoroughly and stirred at room temperature for 20 minutes. The resulting LNPs-LyP-1-*Dab2* mRNA and LNPs-LyP-1-GFP mRNA were further purified by ultrafiltration (MW cutoff, 10 kDa) with 1× PBS (pH7.4) to remove naked mRNAs and ethanol. The final mRNA-loaded LNPs were maintained in 1× PBS at an mRNA concentration of 75 μg/mL (60). DLS was adopted to characterize the LNPs-LyP-1-*Dab2* mRNA and LNPs-LyP-1-GFP mRNA (59, 61). As shown in Supplemental Figure 3B, the average sizes were 136.7 ± 2.52 nm for LNPs-LyP-1-*Dab2* mRNA and 120.5 ± 2.45 nm for LNPs-LyP-1-GFP mRNA, with zeta potentials of –5.54 ± 0.17 mV and –6.11 ± 0.51 mV, respectively.

### Injection of LNPs into mice.

*Dab2* mRNA was loaded into LNPs to facilitate the in vivo application by protecting mRNA from enzymatic degradation, enhancing cellular uptake and endosomal escape, or improving systemic circulation. This encapsulation process was crucial to maintaining the stability and effectiveness of the *Dab2* mRNA for its use in living organisms. The prepared *Dab2* mRNA LNPs (LNPs-LyP-1-*Dab2* mRNA or control LNPs-LyP-1-GFP mRNA) were administered to mice by i.v. injection, with a dosage of 15 μg per mouse. These treatments were given twice a week for a total of 4 weeks.

### Lentivirus-mediated Dab2 overexpression.

Lentivirus for Dab2 overexpression was produced using the PEI STAR transfection method (62–65) in ECs. Initially, ECs were seeded in a 10 cm dish and incubated at 37°C with 5% CO_2_ until they reached 60–70% confluence. For transfection, a mixture containing equimolar amounts of pMD2.G (VSVg) and pSPAX2 (Gag and Pol), along with a double molar amount of pGenLenti *Dab2*-Flag transfer vector, was prepared for a total of 10 μg of DNA. This DNA mixture was combined with 30 μL of PEI STAR (1 mg/mL) in 500 μL of fresh medium, incubated for 10 minutes, and then added dropwise to the cells. After 24 hours, the medium was replaced to remove residual transfection reagent, and the process was repeated after 48 hours. After 72 hours of transfection, the supernatant containing the viral particles was harvested, centrifuged at 500*g* to remove cell debris, filtered through a 0.45 μm filter (Thermo Fisher Scientific), and stored at –80°C for subsequent experiments.

### CRISPR/Cas9-mediated mutations to block FOXM1 binding to the mouse Dab2 promoter.

CRISPR/Cas9-mediated gene editing was used to introduce targeted mutations within the *Dab2* promoter region of mouse skin ECs to disrupt the FOXM1 binding site. The Ad5CMVspCas9/RSVeGFP vector was purchased from the Viral Core at the University of Iowa, using the expression of SpCas9 and a GFP reporter. The selected sgRNA sequences were sgRNA1: TAAGATTCTCTACTATGTG (+ strand); sgRNA2: TTGTATATATCTTGGGGAA (– strand). A 1,154 bp DNA fragment with 3 FOXM1 transcription factor binding sites spans from –4,806 bp to –5,906 bp upstream of the transcription start site of *Dab2*. The synthetic DNA fragment containing mutations in the protospacer adjacent motifs and FOXM1 binding sites was obtained from Synthego. To prevent FOXM1 from binding to the *Dab2* promoter and verify whether cell transduction was successful, the 3 binding sites of FOXM1 were mutated into restriction enzyme sites in the synthetic DNA fragment, as follows: site 1: Sal1 restriction site (AAATGC > GTCGAC); site 2: Fsp1 restriction site (CAATGC > TGCGCA); site 3: Sal1 restriction site (TAATGA > GTCGAC).

For the transduction of ECs, the Ad5CMVspCas9/RSVeGFP vector was introduced using Lipofectamine 3000 (Thermo Fisher Scientific) to maintain the integrity and viability of cells. After 3 days, the synthetic DNA recombination fragment was introduced into the ECs employing the Amaxa Nucleofector 1 Electroporation System and associated kit, strictly adhering to the manufacturer’s protocol. The electroporation conditions were optimized to ensure high viability and efficient uptake of the DNA constructs. After transfection, cells were cultured under standard conditions and screened for GFP expression using fluorescence microscopy. DNA isolated from transduced ECs was then digested with restriction enzymes to further confirm successful delivery and expression of CRISPR components.

### ChIP-qPCR.

Following genomic editing, ChIP-qPCR was performed to verify the impact of mutation on FOXM1 binding to the *Dab2* promoter. The ChIP procedure made use of a kit (Abcam), with cell fixation, chromatin shearing by sonication, and immunoprecipitation with specific antibodies targeting FOXM1. The DNA-protein complexes were pulled down using protein A beads, and the DNA was purified and analyzed by PCR to assess the binding activity of FOXM1 at the modified *Dab2* promoter. The ChIP-qPCR primers of *Dab2* are as follows: forward: 5′-CCCAGCAGTACAAGTCTGGA-3′; reverse: 5′-AGGACTGAGTGGACATGGTG-3′.

### Western blot analysis.

To extract total proteins, cells were lysed using RIPA buffer. Equal amounts of denatured protein were loaded onto a 10% SDS-PAGE gel for electrophoresis. The separated proteins were then transferred to a PVDF membrane and blocked with 5% skimmed milk for 30 minutes at room temperature. Primary antibodies were diluted at 1:1,000, and the secondary antibodies were diluted at 1:2,000. The antibodies are listed in Supplemental Table 3.

### IF staining.

For IF staining, primary mouse ECs were cultured on glass coverslips and fixed in 4% paraformaldehyde for 10 minutes. The cells were then permeabilized with 0.3% Triton X-100 in PBS for 10 minutes and blocked in a solution containing 5% donkey serum in PBS for 1 hour at room temperature. The primary antibodies were diluted in blocking solution and incubated with the cells overnight at 4°C. The next day, the coverslips were washed with PBS and incubated with the respective secondary antibodies conjugated to fluorescent labels (Alexa Fluor) for 2 hours at room temperature. After washing with PBS, the coverslips were mounted on glass slides using VECTASHIELD mounting medium with DAPI (Vector Laboratories) and visualized using a fluorescence microscope (ZEISS LSM 880) with appropriate filters. Images were acquired using a digital camera (ZEISS AxioCam). Unless otherwise specified, secondary antibodies for immunohistochemistry were applied at a concentration of 1:200. A total of 12 images of fields per sample were obtained for the analysis. The antibodies are listed in Supplemental Table 3.

### In vitro wound healing assays.

An in vitro scratch wound–healing assay was performed to evaluate the migration of WT and Dab2-EC^iKO^ cells under normoglycemic and hyperglycemic conditions. Cells were seeded in a 24-well plate and grown to confluence. A scratch wound was made in the cell monolayer using a sterile pipette tip, and the cells were washed to remove any detached cells. The cells were then cultured in either a normoglycemic condition (5 mmol/L glucose) or a hyperglycemic condition (20 mmol/L glucose) in the presence or absence of VEGFA (100 ng/mL), and images were taken at specified time points to monitor cell migration into the wound area. The rate of cell migration was calculated by measuring the width of the wound at different time points. The differences in wound closure between WT and Dab2-EC^iKO^ cells under both normoglycemic and hyperglycemic conditions were analyzed.

### Vascular network formation assays.

An in vitro Matrigel assay was performed to compare the behavior of WT and Dab2-EC^iKO^ cells in normoglycemic and hyperglycemic conditions. The cells were seeded on top of a Matrigel matrix in a 24-well plate and cultured in a normoglycemic media (5 mmol/L glucose) or a hyperglycemic medium (20 mmol/L glucose) for a specified period. The cells were fixed and analyzed to determine morphological changes and quantify cell migration and proliferation. The differences in behavior between the WT and Dab2-EC^iKO^ cells were analyzed under both normoglycemic and hyperglycemic conditions.

### EdU staining.

An EdU staining assay was performed to determine the cell proliferation of WT and Dab2-EC^iKO^ cells under normoglycemic and hyperglycemic conditions. The cells were cultured in either a normoglycemic condition (5 mmol/L glucose) or a hyperglycemic condition (20 mmol/L glucose) and then incubated with EdU, a thymidine analog, for 4 hours in the presence or absence of VEGFA (100 ng/mL). The cells were then fixed and processed for staining, including the addition of a DAPI stain to visualize the nuclei. The EdU-incorporated cells were identified by fluorescence using a specific antibody. The number of EdU-positive cells was counted and compared between the WT and Dab2-EC^iKO^ cells under both normoglycemic and hyperglycemic conditions. The differences in cell proliferation were analyzed and quantified.

### In vivo wound healing assays.

A dermal wound-healing assay was performed to evaluate the role of the *Dab2* gene in the wound-healing process. Two groups of mice, WT and Dab2-EC^iKO^, were used in this study. A standardized wound was created on the dorsal surface of each mouse using a surgical blade. The mice were then monitored for approximately 7 days to evaluate wound healing, including changes in wound size and tissue regeneration. At the end of the study, the mice were sacrificed, and the wound sites were collected for further analysis. The collected tissue was processed for histological analysis, including the use of specific stains to evaluate vascular density during wound closure. The differences in wound healing response among the WT and Dab2-EC^iKO^ mice were analyzed and quantified.

### Matrigel plug assays.

An in vivo Matrigel assay was performed to study the angiogenic response of WT and Dab2-EC^iKO^ mice. Matrigel mixed with 100 ng/mL VEGFA was prepared, and 400–500 μL was implanted s.c. into the backs of both WT and Dab2-EC^iKO^ mice. The mice were then monitored for 7 days to evaluate angiogenic response, including the formation of blood vessels in the implantation site. At the end of the study, the mice were sacrificed, and the implantation sites were collected for further analysis. The collected tissue was processed for histological analysis, including the use of specific stains to visualize blood vessels and quantify angiogenic response. The differences in angiogenic response between the WT and Dab2-EC^iKO^ mice were analyzed and quantified. This assay provided valuable information on the role of the *Dab2* gene in modulating angiogenic response in vivo.

### Dab2 inhibitors predicted by molecular modeling.

To predict the Dab2 signaling inhibitors, docking experiments were performed using the ClusPro 2.0 program (66, 67). The 3-dimensional structure of Dab2 PTB domain (PDB ID: 2LSW) was docked into VEGFR2 kinase domain (PDB ID: 3U6J) to generate the predicted binding models of Dab2:VEGFR2 (Supplemental Table 1). Models with the highest scores and best topologies were selected for the proposed models of the interaction between Dab2 and VEGFR2. In the interaction models, a total of 6 Dab2 inhibitory peptides were identified with good scores based on the molecular modeling (Figure 4, B and C).

### Statistics.

Results were presented as the mean ± SD. The evaluation of differences between groups was performed using 2-tailed *t* test with the demonstration of homogeneity of variance. The 1-way ANOVA was used for multiple comparisons followed by Dunnett’s post hoc analysis, by GraphPad Prism 8. The data shown are representative of 2 or 3 independent experiments. A *P* value of less than 0.05 was considered statistically significant.

### Study approval.

All animal experiments were approved by the Institutional Animal Care and Use Committee at Boston Children’s Hospital. No human study or samples were included.

### Data availability.

RNA-sequencing data are available in National Center for Biotechnology Information Gene Expression Omnibus with the accession number assigned: GSE280770.

## Author contributions

SB and HC conceived the project. SB, JG, YWL, SEB, HW, AEB, JG, SW, YS, JS, and HC participated in experimental design, execution, and data analysis. SB and AEB contributed to mouse corneal micropocket assay. SB, YWL, and HC analyzed the data of bulk RNA sequencing. SB and YWL designed and performed CRISPR/Cas9 experiment. SB and HW performed GTT and ITT assays. YS and JS designed and prepared the nanoparticles. SB prepared lentivirus. SB performed immunohistochemistry and confocal imaging studies. SB, JG, and HW performed Western blot analysis. SB and HW performed the in vitro and in vivo angiogenesis and wound-healing assay. SB and SEB did the quantification and statistics analysis. SB, SEB, JG, DBC, and HC wrote the manuscript. SB, JG, SEB, HW, YWL, KL, WH, WW, JYJS, DBC, MA, JS, and HC reviewed and edited the manuscript.

## Supplementary Material

Supplemental data

Unedited blot and gel images

Supporting data values

## Version 1 01/23/2025

Electronic publication
