# Supplementary material for: Endothelial FOXM1 and Dab2 promote diabetic wound healing
Source: JCI Insight. 2025 Jan 23;10(2):e186504. doi: 10.1172/jci.insight.186504 (PMC11790024; doi:10.1172/jci.insight.186504)
Supplement: Supplemental data [file jciinsight-10-186504-s115.pdf]

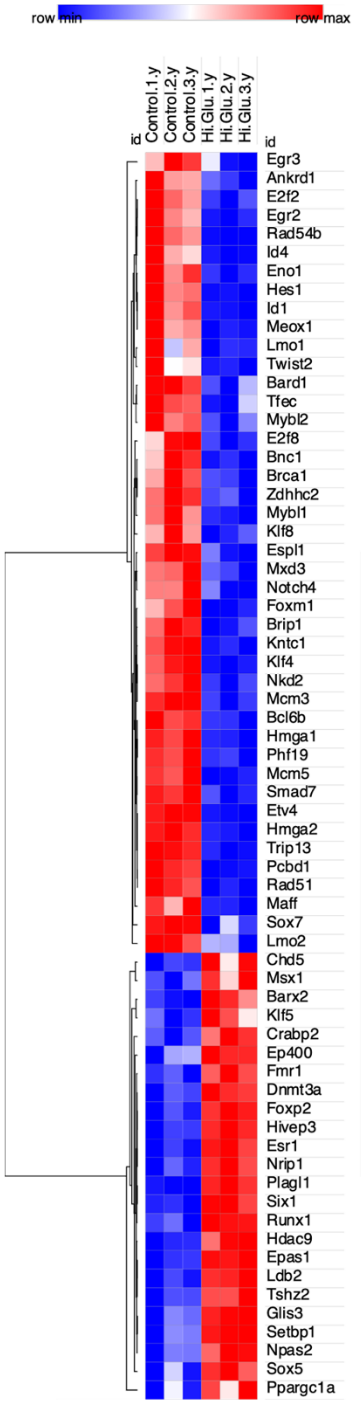

Figure S1. Heatmaps showing differentially regulated gene expression in CD31-enriched primary mouse skin ECs exposed to normal or high glucose concentrations for 48 hours.

Differential gene expression analysis revealed 168 significantly downregulated and 386 significantly up-regulated genes in the ECs grown in high glucose culture conditions compared to ECs cultured in normal glucose media. Sample genes are shown (n = 3 per group of mice).

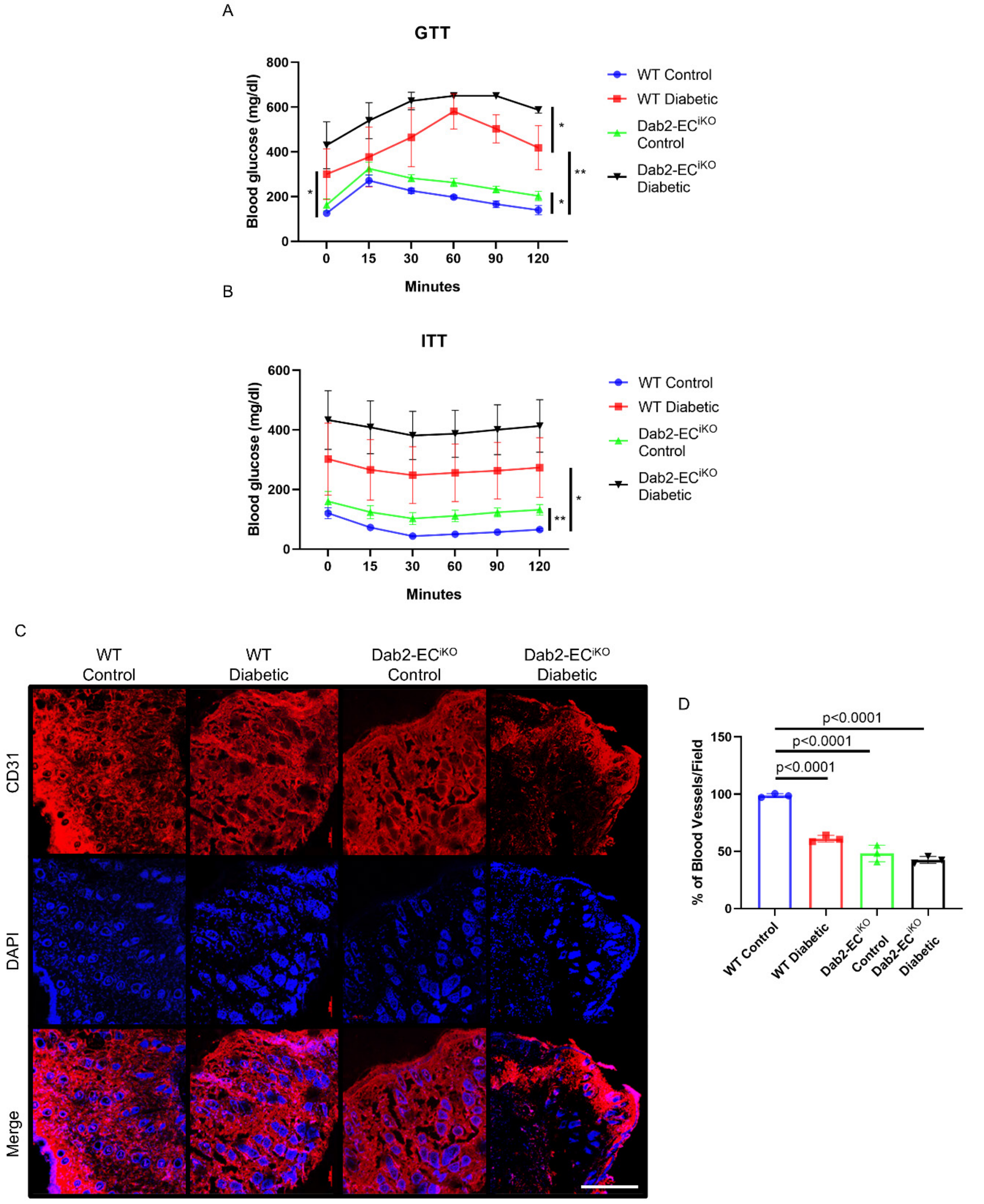

Figure S2. GTT and ITT of HFD WT mice and Dab2-EC^{iKO} mice, and wound CD31 in diabetic WT mice and Dab2-EC^{iKO} mice.

(A) GTT of WT and Dab2-EC^{iKO} mice with or without diabetes after low-dose STZ injection and 12-weeks HFD feeding described in Figure 2E. (n = 3-6 per group of mice, results are presented as mean ± SD, p value calculated by Student's t-test, *p<0.05, **p<0.01).

(B) ITT of WT and Dab2-EC^{iKO} mice with or without diabetes after low-dose STZ injection and 12-weeks HFD feeding described in Figure 2E. (n = 5 per group of mice, results are presented as mean ± SD, p value calculated by Student's t-test, *$p<0.05$, **$p<0.01$).

(C) Representative immunofluorescence staining of CD31 (red) in wound area from collected in mice described in Figure 2D. Scale bar=100μm.

(D) Quantitation of CD31-positive blood vessel density in Figure 2D. (n = 3 per group of mice, results are presented as mean ± SD, p value calculated by ANOVA).

A

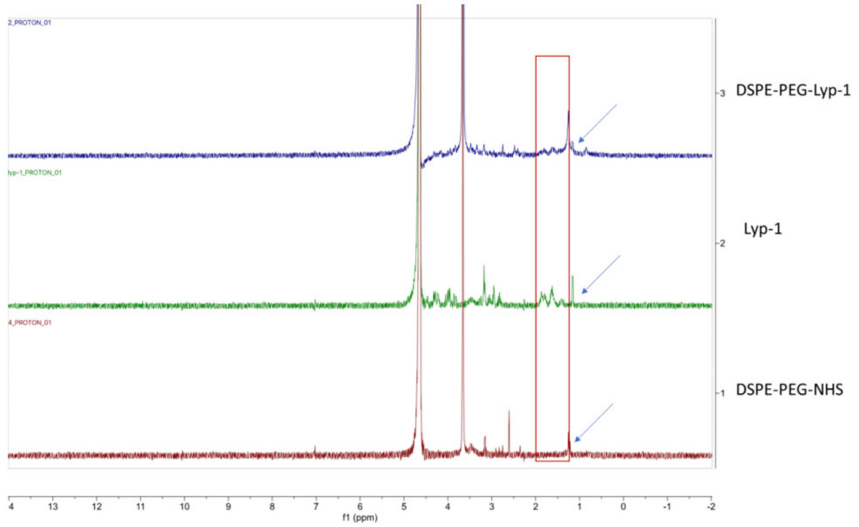

B

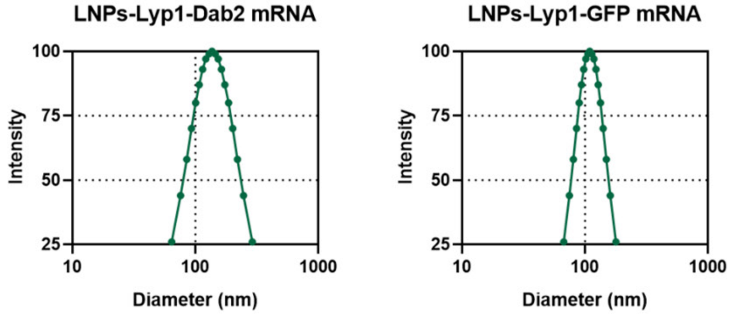

LNPs	Size-diameter (nm)	Zeta-potential (mV)
LNPs-Lyp1-Dab2 mRNA	136.7 ± 2.52	-6.11 ± 0.38
LNPs-Lyp1-GFP mRNA	120.5 ± 2.45	-5.54 ± 0.17

Figure S3. Characterization of the Lyp1-LNP-*Dab2* mRNA- and Lyp1-LNP-GFP mRNA-containing lipid nanoparticles (LNPs).

(A) ^{1}H-NMR to characterize the successful synthesis of DSPG-PEG-Lyp1.

(B) Size and Zeta potential of LNPs-Lyp1-*Dab2* mRNA and LNPs-Lyp1-GFP mRNA (control group).

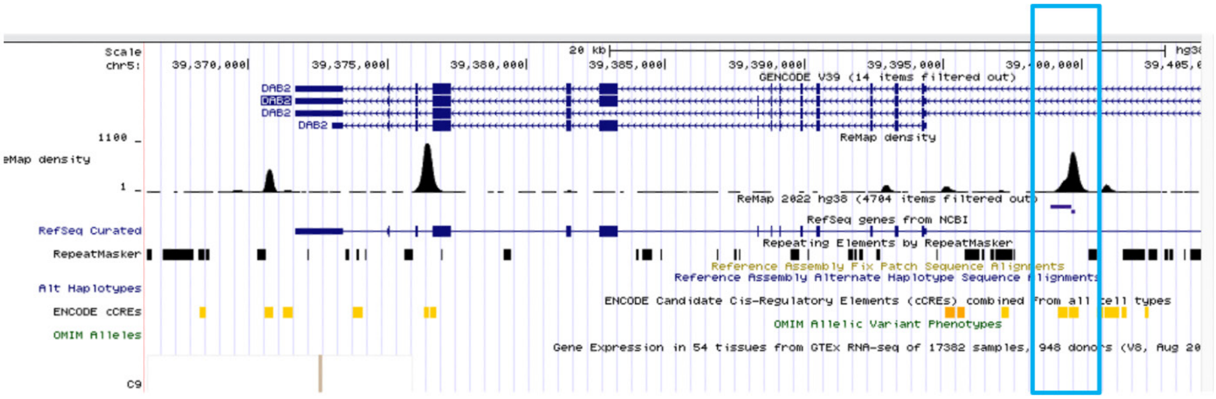

Figure S4. FOXM1 binding zone in *Dab2* promoter.

UCSC genome viewer display of FOXM1 binding sites (boxed peak) in *Dab2* promoter region. The x-axis represents the genomic region of the *Dab2* promoter, the y-axis shows the peak height of the FOXM1 binding sites.

Supplemental Table 1. Interacting residues between mouse Dab2 (2LSW) and VEGFR2

The crystal structure of mouse Dab2 (PDB ID: IP3R) and VEGFR2 were taken from the PDB database and used to perform the docking experiments using ClusPro 2.0. There are total 200 models per each docking experiment. The frequency of residues in Dab2 and VEGFR2 to form H-bonds among these models were ranked, respectively. From the modeling, the E33, K49, Q153, E37, and Q88 residues from Dab2 and the R1027, K1023, R1126, D1129, R819, R1022, H816, and Y996 residues from VEGFR2 were critical to form the complex.

Interaction residues of Fibromodulin to form H-bonds		Interaction residues of AXL to form H-bonds	
Predicted residues in Dab2(IP3R)	Frequency to form H-bonds	Predicted residues in VEGFR2	Frequency to form H-bonds
E33	21	R1027	27
K49	17	K1023	15
Q153	17	R1126	15
W37	16	D1129	14
M32	16	R819	13
Q88	12	R1022	11
E156	11	H816	11
K108	11	Y996	11
R42	10	R1172	10
N131	10	E815	9
Q154	10	K826	9
Y38	9	1170	9
R92	9	R1080	9
Q86	9	D1046	8
R132	9	N933	8
K44	8	H1173	7
T35	8	H891	6
H89	7	R932	6
R84	7	Q1149	6
E113	7	E1146	5
K108	7	E1134	5
I57	7	D1171	5
R126	6	Y1082	5
D106	5	Q1137	4
Q91	5	935	4
D164	5	L995	4
T109	5	Y1130	4

D59	4	R1061	3
D161	4	D1028	3
Y50	4	E993	3
K34	4	R1124	3
E115	4	D1141	3
Q145	4	D994	3
Q143	4	D823	3
K163	3	R880	3
H114	3	I1025	3
D66	3	G1122	3
K90	3	H1159	2
D46	3	E1155	2
K51	3	Q1085	2
T129	3	E1158	2
G87	3	Y1054	2
S85	3	R1066	2
K53	2	H894	2
K75	2	Y938	2
K77	2	Y1136	2
K150	2	R1118	2
R64	2	R1022	2
Q91	2	Y822	1
Q167	2	H1026	1
		D1064	1
		R1051	1
		R1052	1
		K1120	1
		E815	1
		E1017	1
		R1051	1
		R1052	1
		K997	1
		K1070	1

Supplemental Table 2. List of qRT-PCR primers.

Gene	Forward primer	Reverse primer
Kdr (VEGFR2)	5'- ATCCACTGGTATTGGCAGT - 3'	5'- AGGTGCCCAGGAAAAGACGA - 3'
Dab2	5'- CCCAGCAGTACAAGTCTGGA - 3'	5'- AGGACTGAGTGGACATGGTG - 3'
FoxM1	5'- ACCATAGCAACCCTAGCAGC - 3'	5'- GGGTACCACAGGATGAAAGCA - 3'
Ets1	5'- ACGCTGCATCCTATCAGCTC - 3'	5'- CGAGTTTACCACGACTGGCT - 3'
GATAd1	5'- GCAAGATGGGAAGCCGTACT - 3'	5'- GACTGGCGAGGGTAGGAATG - 3'
GAPDH	5'- GTCTCCTCTGACTTCAACAGCG - 3'	5'- ACCACCCTGTTGCTGTAGCCAA - 3'
B-Actin	5'- AGAGCTACGAGCTGCCTGAC - 3'	5'- AGCACTGTGTTGGCGTACAG - 3'

Supplemental Table 3. List of antibodies.

Target antigen	Vendor or Source	Catalog #	Applications	Source Isotype	Cross activity
VEGFR2	Cell Signaling	9698	WB	Rabbit	H M R
phospho-VEGFR2	Cell Signaling	2478	WB/IF	Rabbit	H M
ERK	Cell Signaling	4695	WB	Rabbit	H M R
phospho-ERK	Cell Signaling	9106	WB	Mouse	H M R
Akt	Cell Signaling	9272	WB	Rabbit	H M R
phospho-Akt	Cell Signaling	4058	WB	Rabbit	H M R
Dab2	Santa Cruz	sc-136964	WB/IF	Mouse	H M
FoxM1	GeneTex	GTX100276	WB/IF	Rabbit	H M
Actin	Santa Cruz	sc-58673	WB	Mouse	H M R
GAPDH	Santa Cruz	sc-137179	WB	Mouse	H M R
Anti-Mouse IgG (H+L) Secondary Antibody, HRP	Thermo Fisher Scientific	31430	WB	Goat	M
Anti-Rabbit IgG (H+L) Secondary Antibody, HRP	Thermo Fisher Scientific	31460	WB	Goat	R
CD31	Thermo Fisher	BDB550274	IF	Rat	H M R
Alexa Fluor 488 anti-Rat (H+L)	Invitrogen	A-21208	IF	Donkey	Rat
Alexa Fluor 488 anti-Mouse (H+L)	Invitrogen	A-21202	IF	Donkey	M
Alexa Fluor 488 anti-Rabbit (H+L)	Invitrogen	A-21206	IF	Donkey	R
Alexa Fluor 594 anti-Rat (H+L)	Invitrogen	A-21209	IF	Donkey	Rat
Alexa Fluor 594 anti-Mouse (H+L)	Invitrogen	A-21203	IF	Donkey	M
Alexa Fluor 594 anti-Rabbit (H+L)	Invitrogen	A-21207	IF	Donkey	R
