# Supplementary figures and images for: Endothelial FOXM1 and Dab2 promote diabetic wound healing
Source: JCI Insight. 2025 Jan 23;10(2):e186504. doi: 10.1172/jci.insight.186504 (PMC11790024; doi:10.1172/jci.insight.186504)

Fig 1D

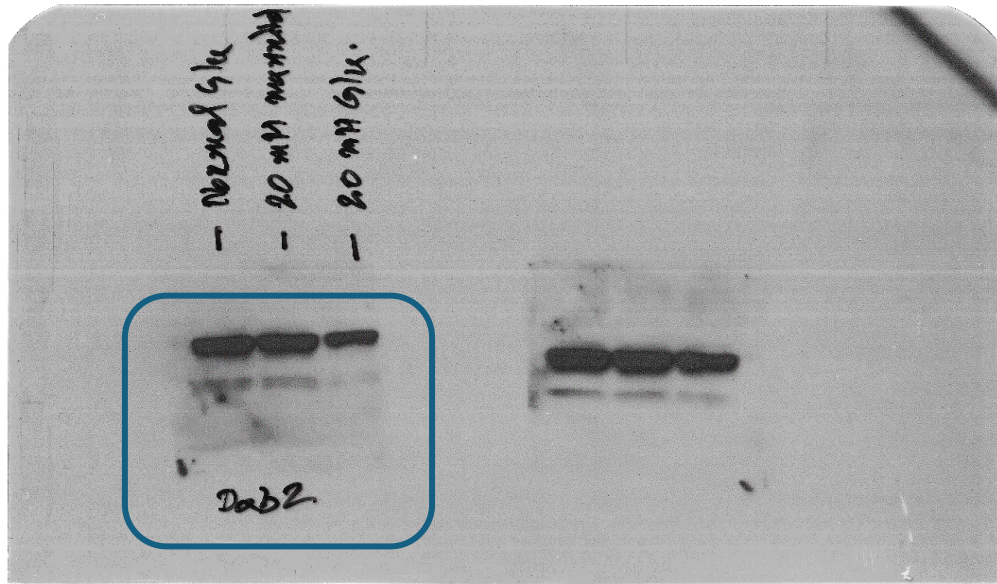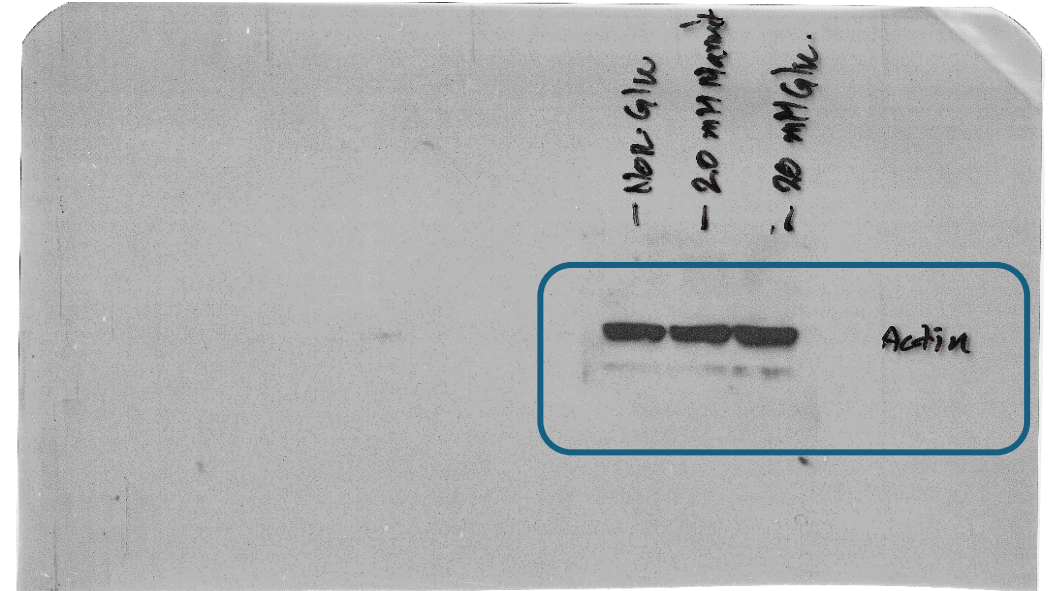

Fig 1F

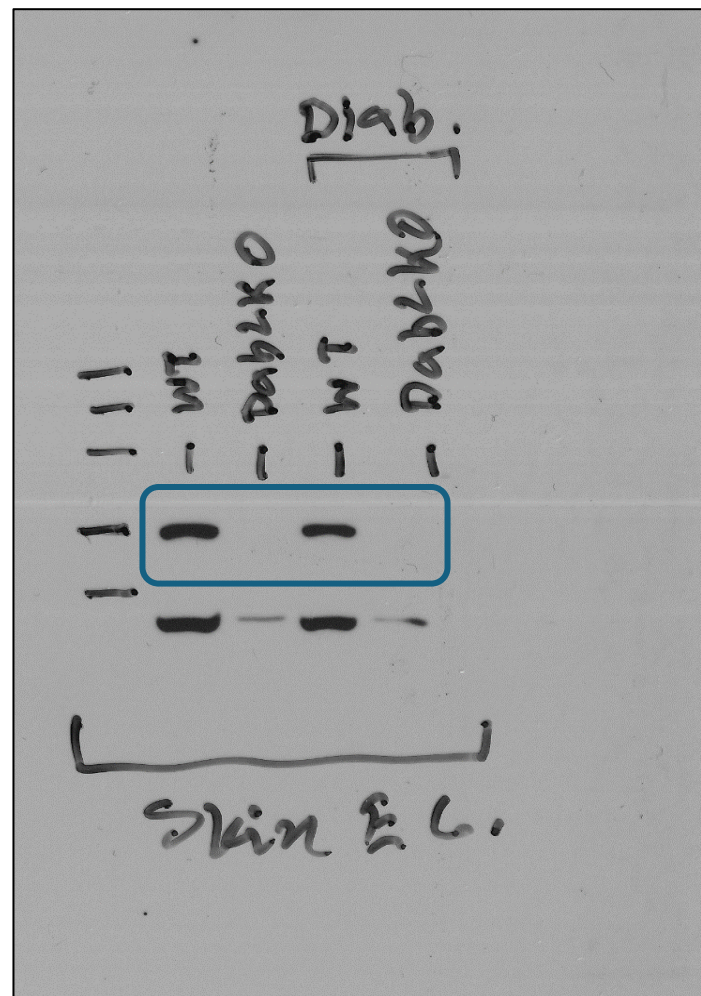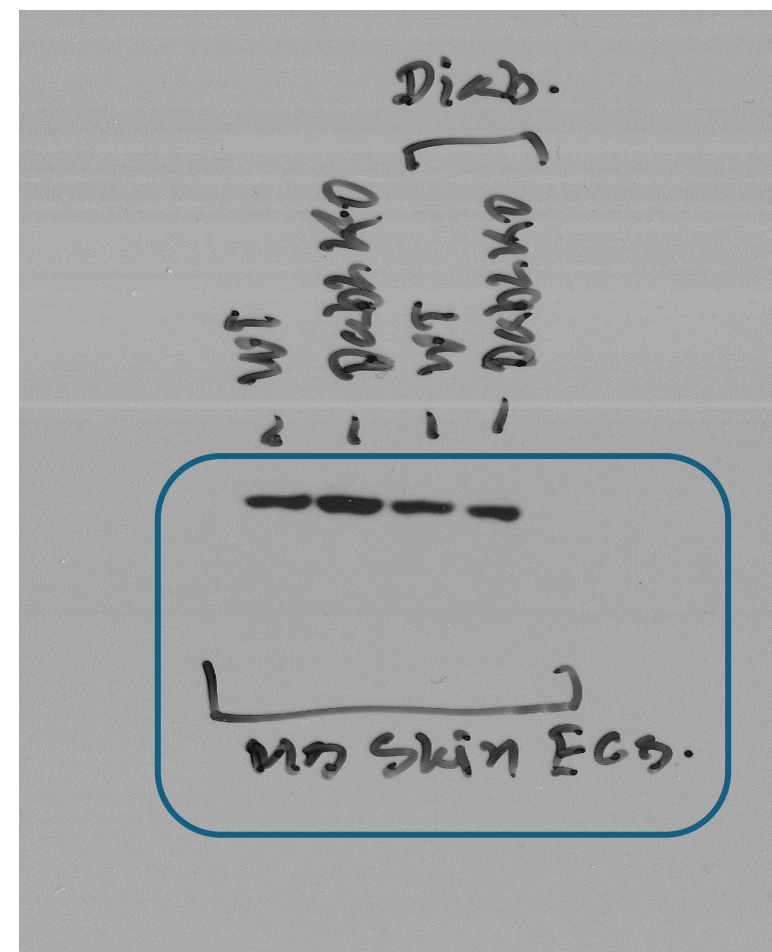

Fig 3I

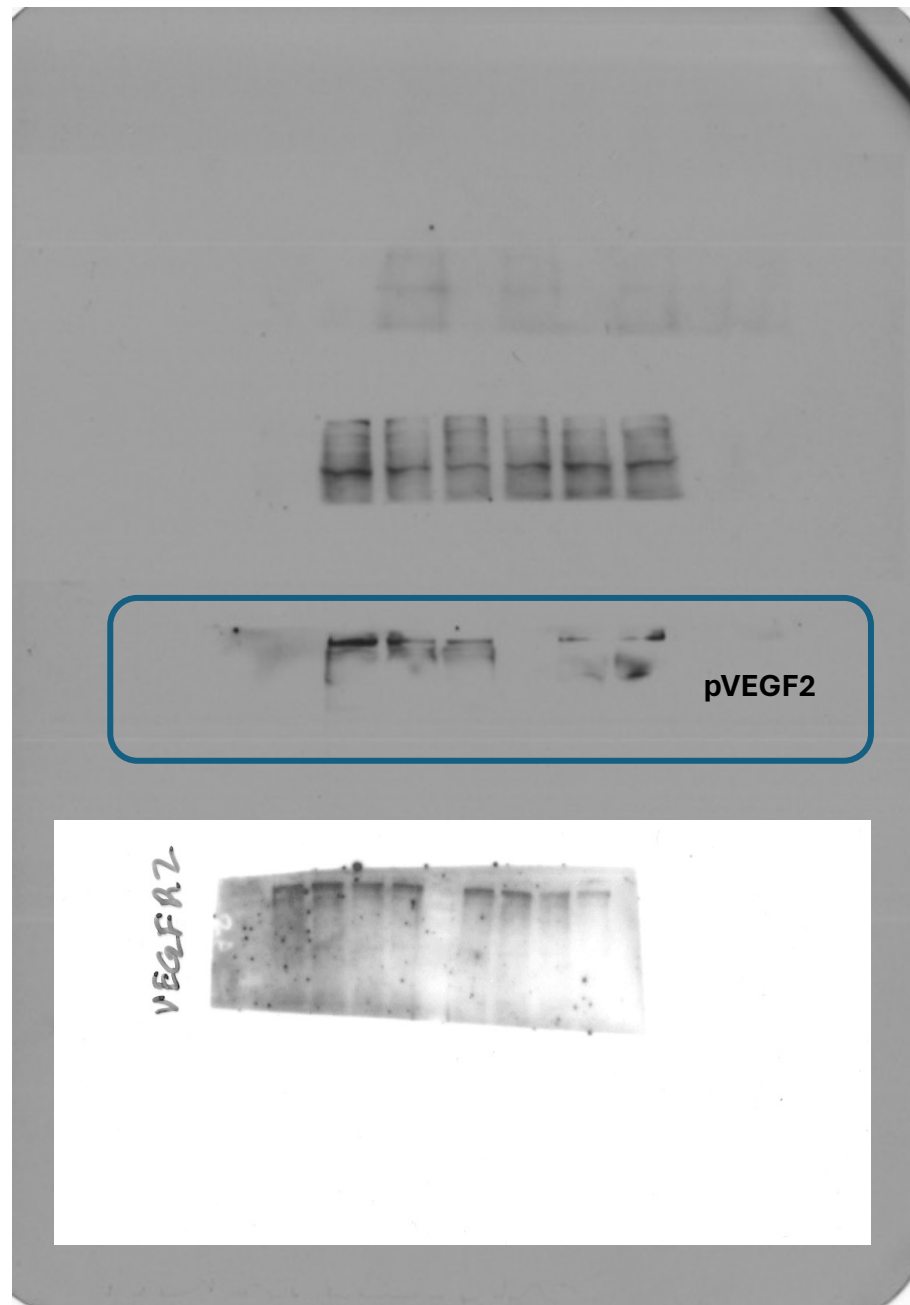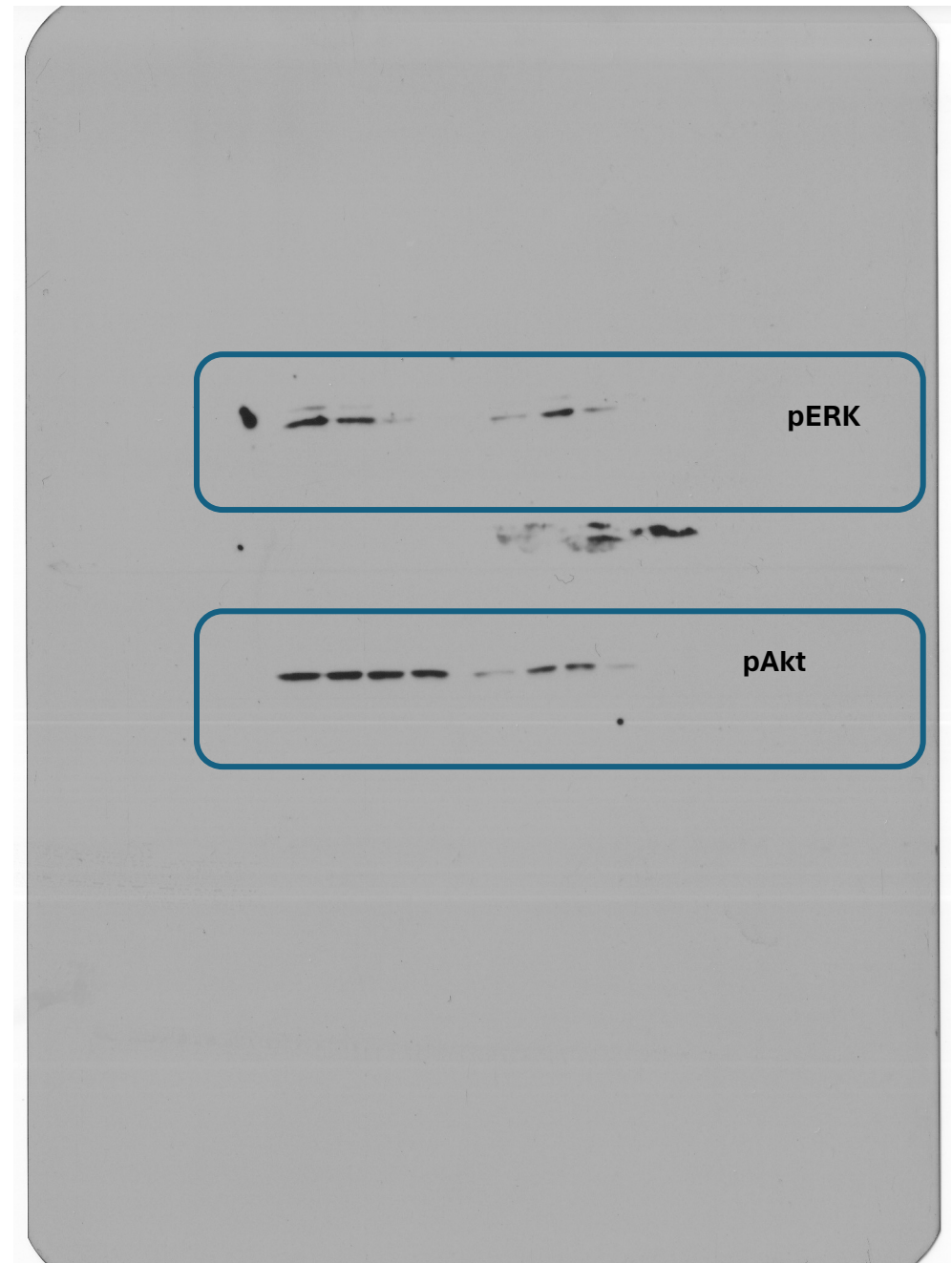

Fig 3I

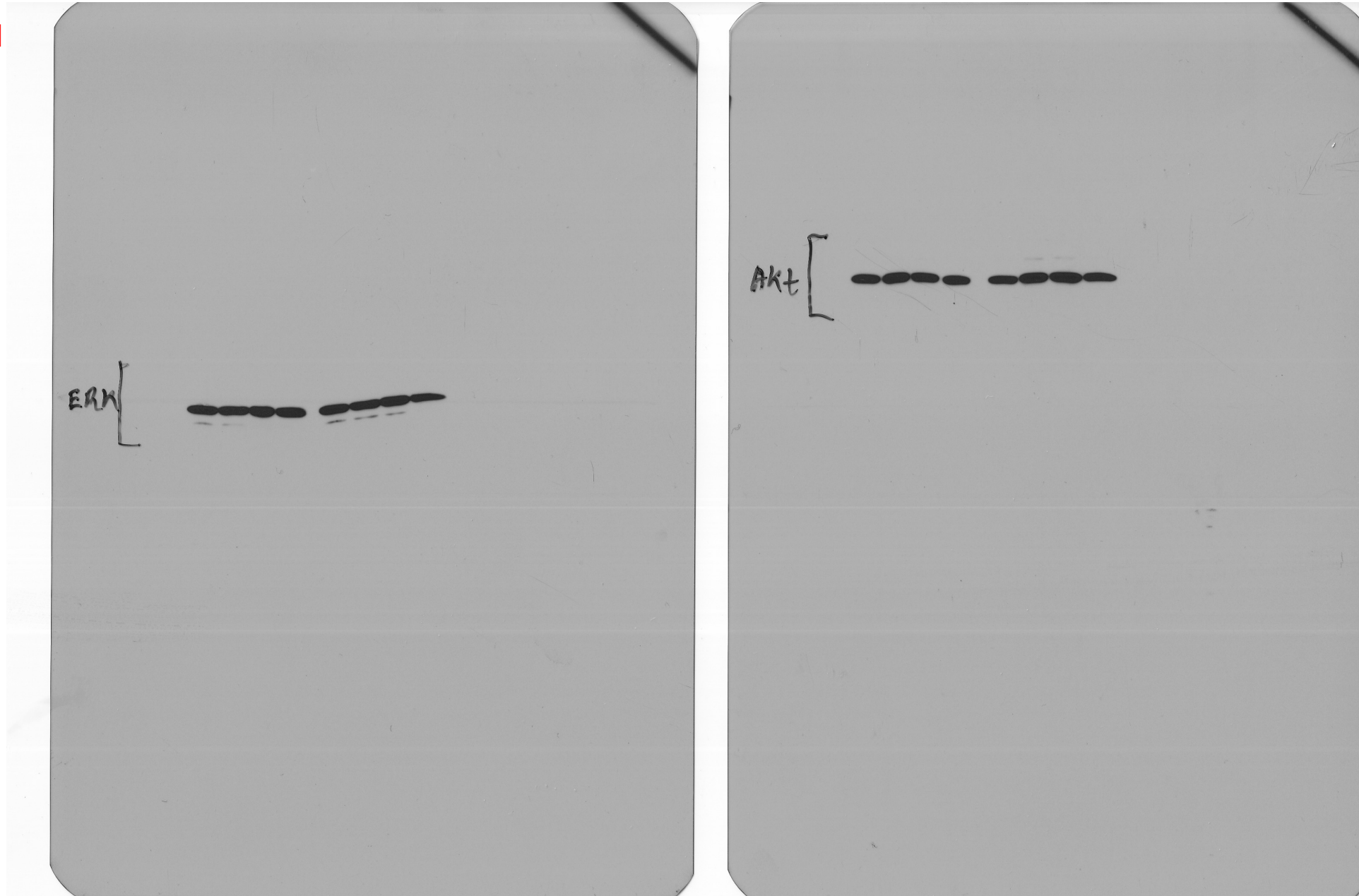

Fig 3I

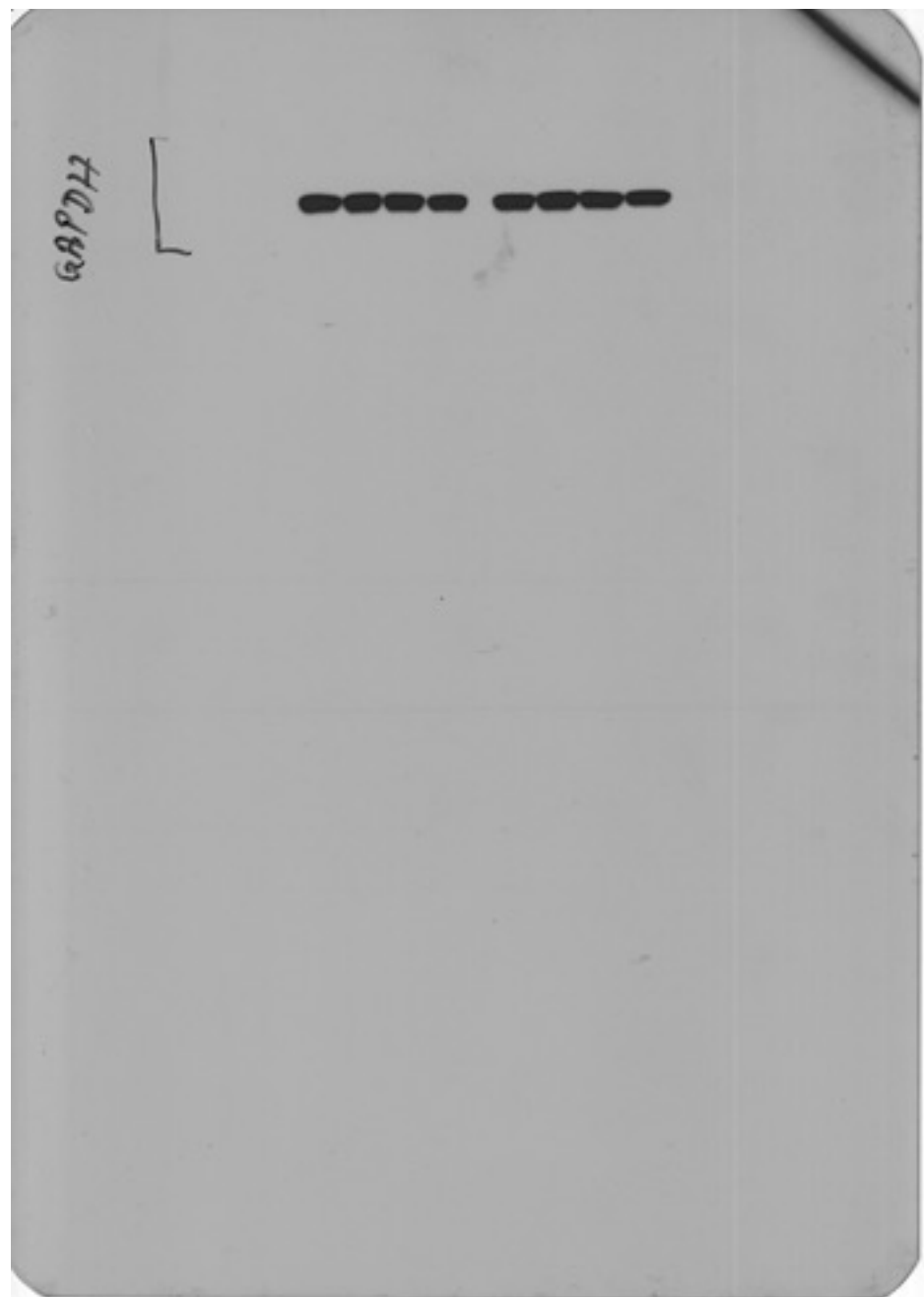

Fig 5D

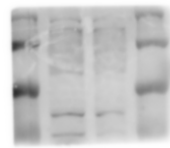

FOXM1

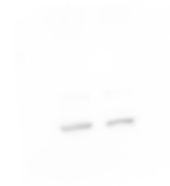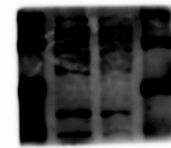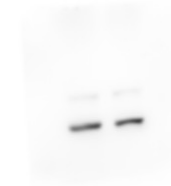

actin

Fig 5F

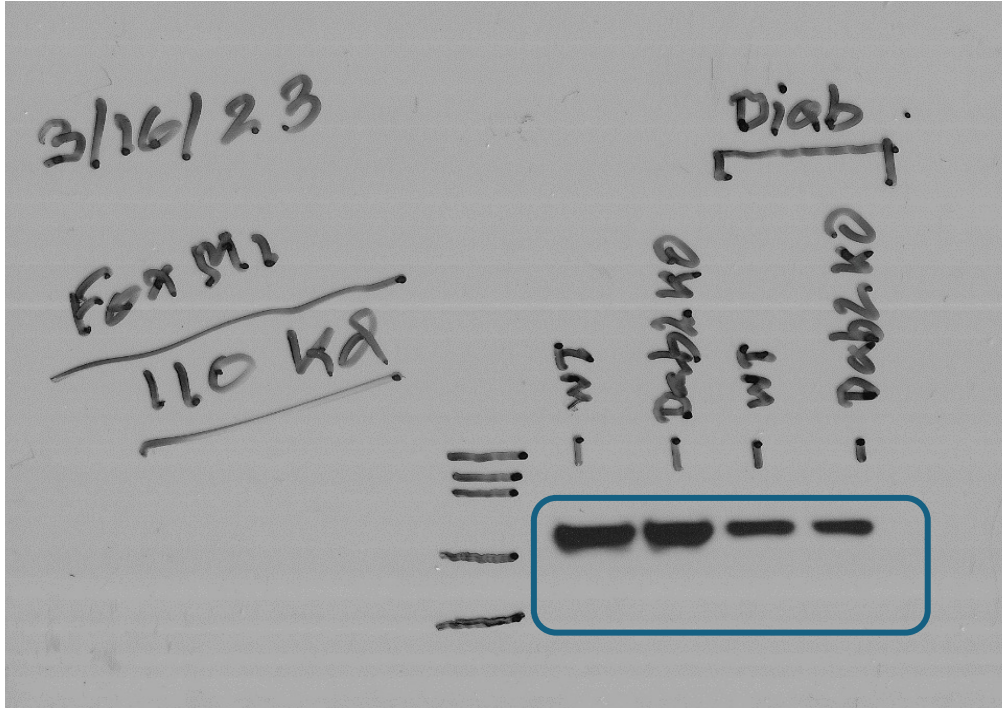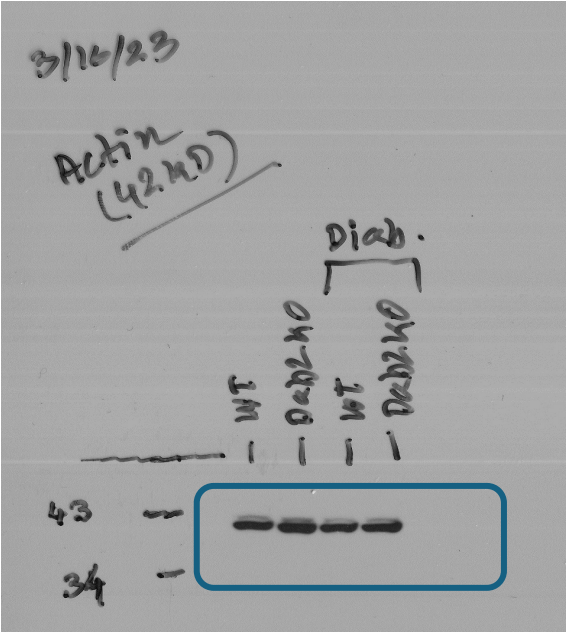

Fig 6D

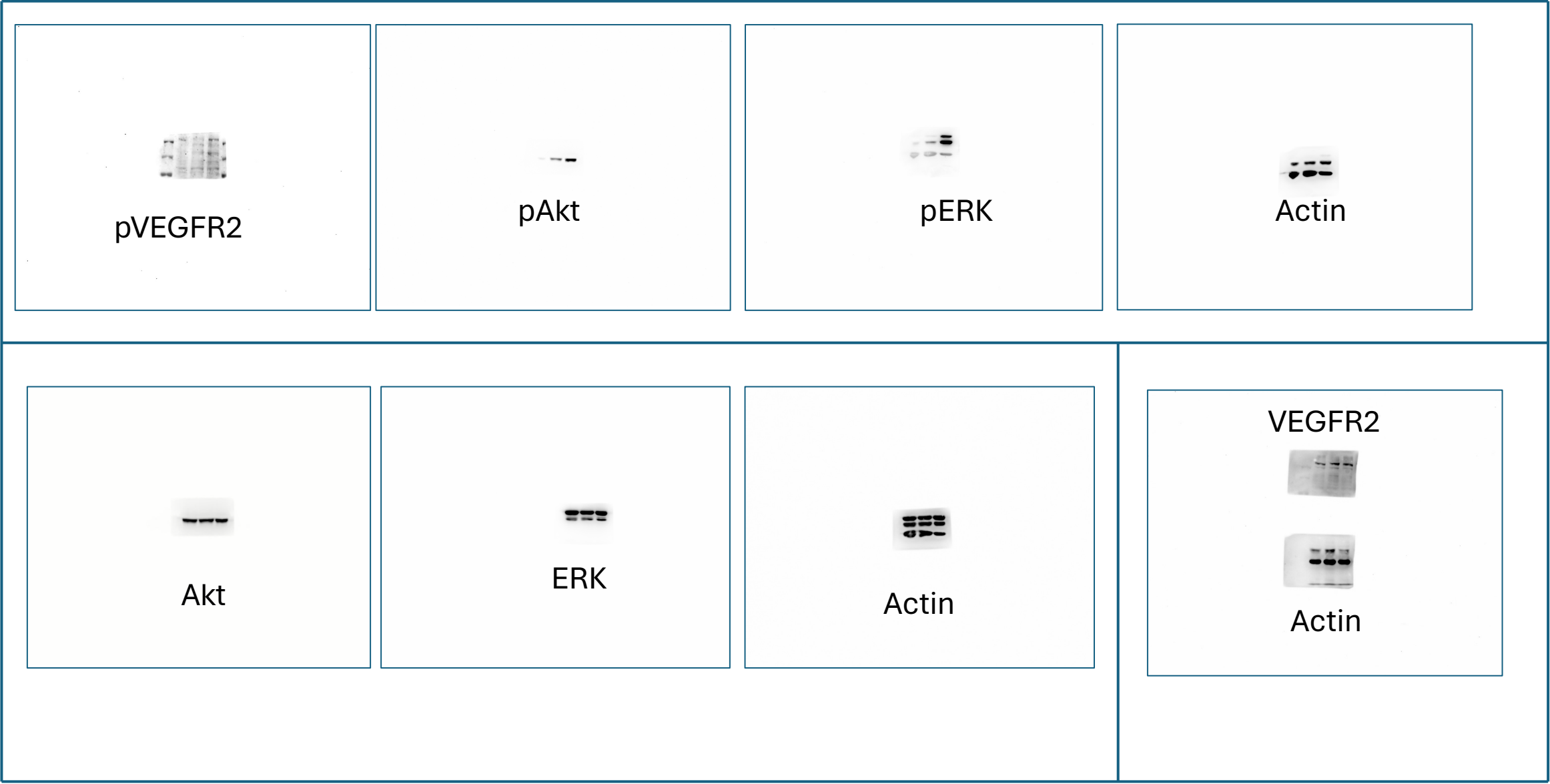

Fig 6F

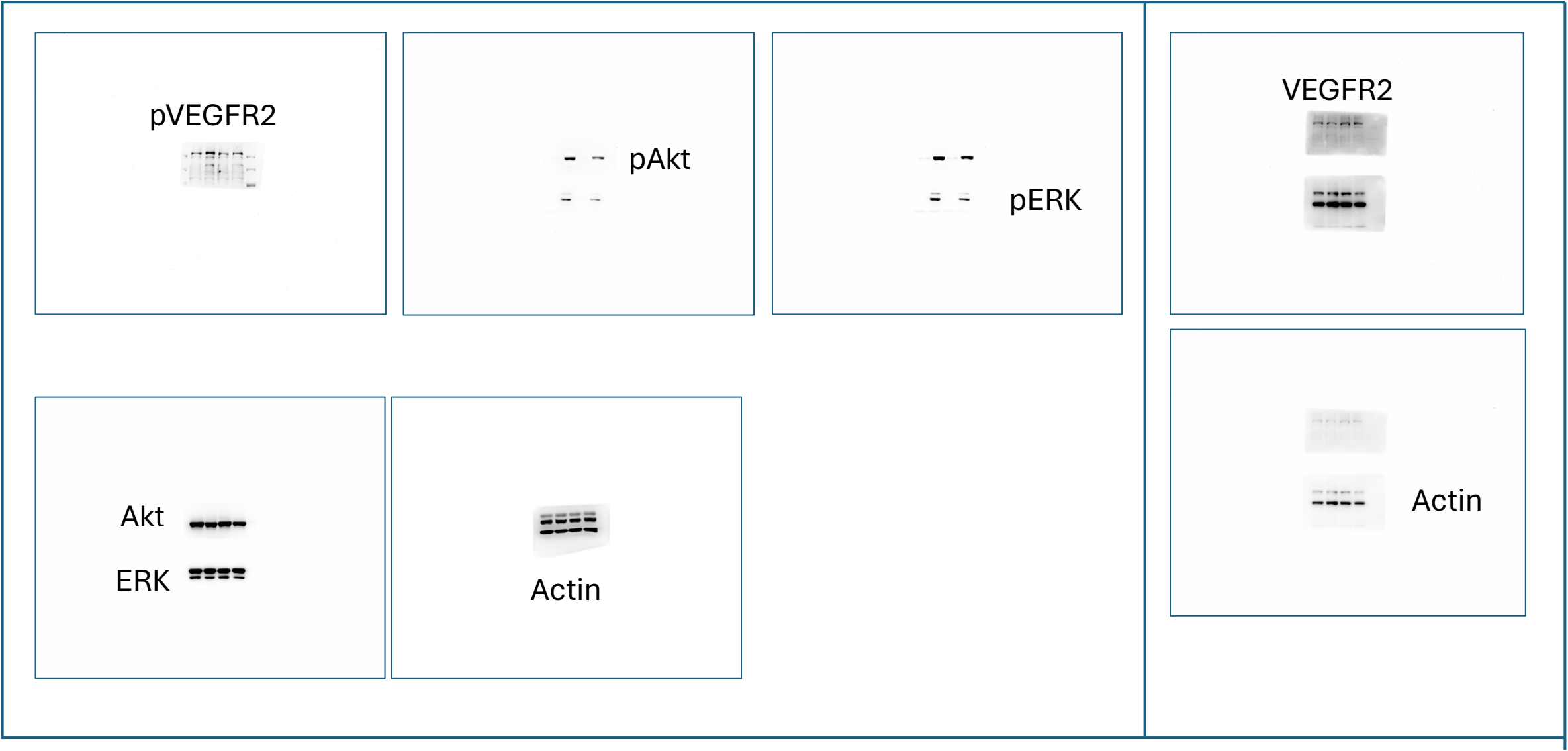

Supplement: Unedited blot and gel images [file jciinsight-10-186504-s116.pdf]
